# In-Depth Analysis of the Re-Emergence of Respiratory Syncytial Virus at a Tertiary Care Hospital in Germany in the Summer of 2021 after the Alleviation of Non-Pharmaceutical Interventions Due to the SARS-CoV-2 Pandemic

**DOI:** 10.3390/v15040877

**Published:** 2023-03-29

**Authors:** Mario Hönemann, Stephanie Thiem, Sandra Bergs, Tom Berthold, Christian Propach, Manuela Siekmeyer, Armin Frille, Tillmann Wallborn, Melanie Maier, Corinna Pietsch

**Affiliations:** 1 Virology Department, Institute of Medical Microbiology and Virology, University of Leipzig, Johannisallee 30, 04103 Leipzig, Germany; 2 Department of Pediatrics, University of Leipzig, Liebigstrasse 20a, 04103 Leipzig, Germany; 3 Department of Respiratory Medicine, University of Leipzig, Liebigstrasse 20, 04103 Leipzig, Germany; 4 Department of Pediatrics, Klinikum St. Georg, Delitzscher Strasse 141, 04129 Leipzig, Germany

**Keywords:** RSV, molecular epidemiology, respiratory infections, respiratory viruses, non-pharmaceutical interventions

## Abstract

Following the extensive non-pharmaceutical interventions (NPIs) and behavioral changes in the wake of the SARS-CoV-2 pandemic, an interseasonal rise in respiratory syncytial virus (RSV) cases was observed in Germany in 2021. The aim of this study was to characterize the local molecular epidemiology of RSV infections in comparison to the three pre-pandemic seasons. Additionally, clinical data were retrieved from patient charts to determine the clinical significance of RSV infections. RSV detections peaked in calendar week 40 of 2021, 18 weeks earlier than the usual peak observed in the three pre-pandemic seasons. Sequence analysis revealed a close phylogenetic relatedness regardless of the season of origin. A significantly higher amount of pediatric cases (88.9% of all cases, *p* < 0.001) was observed for season 2021/2022. For the pediatric cases, significant differences were observed for an increased number of siblings in the household (*p* = 0.004), a lower rate of fever (*p* = 0.007), and a reduced amount of co-infections (*p* = 0.001). Although the mean age of the adult patients was significantly younger (47.1 vs. 64.7, *p* < 0.001), high rates of comorbidities, lower respiratory tract infections and intensive care unit admissions prevailed. The NPIs in the wake of the SARS-CoV-2 pandemic had a tremendous impact on the epidemiologic characteristics and seasonality of RSV and warrant further epidemiologic studies of this important pathogen.

## 1. Introduction

Respiratory syncytial virus (RSV) is one of the most widespread respiratory pathogens and affects all age groups across the population [1]. In addition to mild upper respiratory tract infections (URTI), RSV can also cause severe lower respiratory tract infections (LRTI), especially in the form of obstructive bronchiolitis. RSV infections are among the most common reasons for hospitalization in infants and young children below the age of two years [2]. Particularly, premature infants are at high risk for severe courses of disease. A major reason for this is the high surface-to-volume ratio of individual bronchioles, which represents a predisposition for airway obstruction subsequent to reversible immune-mediated epithelial damage, mucosal edema, and hypersecretion of the still-developing airways [3,4]. RSV infections of adults seem to predominantly occur in the form of mild URTI [5]. However, comorbidities or co-factors such as immunosuppressive drugs, e.g., in the context of solid organ transplantation, may provide an increased susceptibility to infections with the risk for severe disease progression [6,7]. In Europe, infections typically occur in late autumn and the winter months with a peak in February [8].

RSV is a negative-stranded RNA virus belonging to the genus *Orthopneumovirus* in the family *Pneumoviridae* and can be subdivided into two subtypes, RSV-A and RSV-B, based on genetic and antigenic diversity [8,9]. The virion contains a lipid envelope with embedded glycoproteins, most notably the fusion protein (F) and the attachment glycoprotein (G). The G protein initiates cell binding, while the F protein mediates fusion of the virion with host cells. The F protein is the main target for vaccine development and the target of the monoclonal antibodies palivizumab and nirsevimab, which may be administered as a prophylaxis to children [10,11]. According to the genetic variability of the *G gene*, RSV subtypes can be further subdivided into several genotypes [12,13]. Although the complete sequence of the *G gene* or full genome sequences provide the highest resolution, recent proposals for revised genotype descriptions identified the G ectodomain as the lowest common denominator suitable for RSV genotyping [14,15,16].

In addition to behavioral changes, extensive non-pharmaceutical interventions (NPIs) [17], which included nationwide lockdowns with the subsequent closing of schools, daycare centers, and cultural events, were implemented in Germany in 2020 due to the SARS-CoV-2 pandemic. Concomitantly, RSV activity was greatly reduced during winter 2020/2021. Following the alleviation of NPIs, an interseasonal rise in RSV cases was observed in the summer and fall of 2021. The aim of this study was to characterize the local molecular epidemiology of RSV infections and the age distribution of the patient population between seasons 2017/2018, 2018/2019, and 2019/2020 before the SARS-CoV-2 pandemic and the season of 2021/2022. Additionally, the clinical significance of the RSV cases was analyzed based on the clinical data for the same patient group.

## 2. Materials and Methods

### 2.1. Sample Collection and Clinical Data

From October 2017 to September 2022, 19,734 respiratory samples from 4549 pediatric (<18 years) and 8042 adult (≥18 years) in- and outpatients were collected and tested for viral respiratory infections at the University Hospital of Leipzig, Germany. Samples included nasal and/or naso-oropharyngeal swabs (56.7%, *n* = 11,204), throat rinsing fluids (23.1%, *n* = 4552), bronchoalveolar lavage fluids (10.9%, *n* = 2154), tracheal secretions (7.4%, *n* = 1459), nasal secretions (1.2%, *n* = 228), and sputa (0.8%, *n* = 137). Testing was initiated at the discretion of the attending physician. To avoid a bias caused by follow-up samples, re-testing within six weeks after the initial detection was defined as a single case. Data relating to underlying medical conditions and clinical parameters from the day of RSV detection were retrieved retrospectively from patient charts. In the case of missing clinical information, the designation “(*n*/total)” indicates the respective cases for the total amount of available data. A body temperature >38.0 °C was categorized as fever. The classification of URTIs and LRTIs was carried out according to the International Statistical Classification of Diseases and Related Health Problems (ICD-10-WHO) and the diagnoses and information listed in the patients’ records. Bronchiolitis and bronchitis were classified within the same category. Patients with any of the following conditions were considered immunocompromised: receiving active chemotherapy for cancer, severe chronic neutropenia, receiving steroids, or other immunomodulatory medications over a prolonged period, aplasia of the thymus gland, or reported HIV infection. The RSV infection of inpatients was classified as nosocomial when respiratory symptoms were not present at the time of hospital admission and the beginning of symptoms occurred at least 72 h after admission, or readmission with respiratory tract symptoms within 48 h after discharge. Bacterial and fungal pathogens were cultivated with standard microbiological techniques and considered as co-infections if a detection from respiratory samples or blood was documented. Viral co-infections were assessed through a multiplex test for respiratory viruses (see below) and through established routine laboratory protocols for HCMV [18,19], EBV [20], and HHV-6 [21]. SARS-CoV-2 was analyzed with a commercially available SARS-CoV-2 RT-PCR assay for the Alinity m analyzer (Abbott, Chicago, IL, USA) targeting the viral *RdRp-* and *N-genes*. RSV seasons before the start of the SARS-CoV-2 pandemic in 2020 were defined as starting on 1 October and ending on 30 September of the following year. Deviating from this definition, the RSV season after the alleviation of NPIs in the spring and summer of 2021 was deemed season 2021/2022.

### 2.2. Meteorological Data

The data for the mean monthly air temperatures (Leipzig/Halle; Station ID: 2932) were obtained from the Climate Data Centre (CDC) of the Deutscher Wetterdienst (DWD) [22].

### 2.3. Nucleic Acid (NA) Extraction and RSV Detection

Total NA was extracted from 200 μL of the respiratory samples using the DNA and Viral NA Small Volume Kit on a MagNA Pure 96 instrument (both, Roche, Mannheim, Germany) according to the manufacturer’s instructions. NAs were stored in aliquots at −80 °C until further use. The presence of genomes of common respiratory viruses, including influenza viruses A and B, RSV-A and -B, parainfluenza viruses 1 to 4, human coronaviruses (including 229E, NL63, OC43, and HKU1), human metapneumoviruses, adeno- viruses, human bocaviruses, rhinoviruses, and enteroviruses, was assessed using a multiplex panel assay (NxTAG RPP, Luminex corporation, Austin, TX, USA) according to the manufacturer’s instructions. Samples that reacted to either one or both RSV targets of the assay were analyzed further.

### 2.4. RSV Genotyping and Phylogenetic Analysis

The complete viral attachment glycoprotein gene (*G gene*) was amplified using a modified protocol based on Ma et al. [12]. The amplicon sizes were approximately 1300 bp for both RSV-A and RSV-B after the final nested PCR step. In cases of low RSV concentration, an alternate protocol generating two overlapping fragments was used. For a detailed description of the reaction conditions and primers, see Supplementary Tables S1–S7. Sanger Sequencing was performed using the BigDye Terminator Sequencing Kit v1.1 and an ABI 3500 Genetic Analyzer (both Applied Biosystems, Foster City, CA, USA). The genotyping approach was applied to the samples of every season 2021/2021 case and to randomly selected samples of at least 10% of the cases of the three pre-pandemic seasons. The obtained sequences were submitted to GenBank (accession numbers OP927816 to OP928144). Separate phylogenetic trees for RSV-A and RSV-B were constructed at the nucleotide level using MEGA software, version 7, based on the maximum likelihood method. Bootstrap analysis was performed with 1000 replicates [23]. For the genotype assignment, separate phylogenetic trees were constructed using (partial) *G gene* reference sequences proposed by Goya et al. [14] and Muñoz-Escalante et al. [15,16] (Supplementary Tables S8–S11). For the analysis of the relatedness of clinical isolates of the current study, two phylogenetic trees for RSV-A and RSV-B were constructed using the complete *G gene* including derived full-length glycoprotein gene consensus sequences of the above proposed genotypes.

### 2.5. Statistical Analysis

Statistical analyses were performed using IBM SPSS Statistics for Windows, version 27.0 (IBM Corp., Armonk, NY, USA). Continuous values were expressed as means or medians (range) and categorical data as frequencies (percentages). A Mann–Whitney U test was performed to compare continuous variables. A Chi-square test was performed for categorical variables. Brackets indicate parameters that were analyzed in the same contingency table. All tests were two tailed. A *p*-level of <0.05 was considered significant. For post hoc pairwise comparisons of column proportions, a Bonferroni correction for multiple comparisons was applied.

## 3. Results

### 3.1. RSV Detection and Seasonality

**Figure 1:**
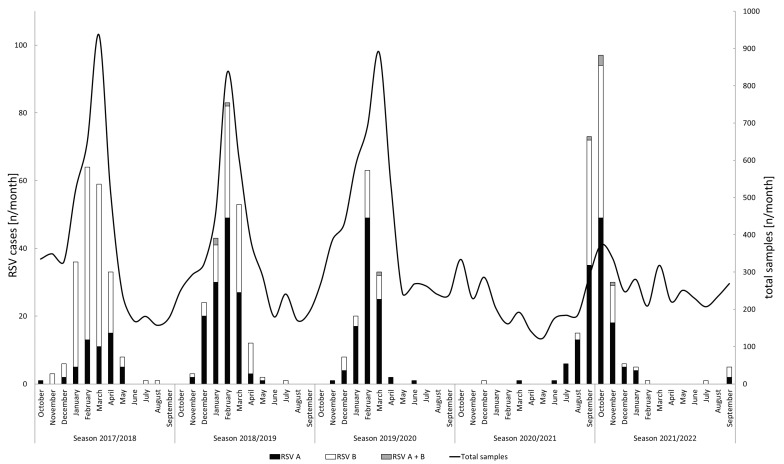
Monthly total numbers of tested samples and RSV cases (*n* = 803) stratified by subtype. Note the two different y-axes: the left axis refers to the bar charts and shows the absolute number of detected RSV cases while the right axis refers to the absolute number of tested samples, as represented by the line graph. The x-axis is labeled according to the pre-pandemic definition that was used to define the start and end of a respiratory season.

In total, the presence of RSV RNA was confirmed in 908 samples originating from 803 unique cases by RT-PCR. The absolute number of RSV cases detected between 2017 and 2022 stratified by subtype are shown in Figure 1. In season 2017/2018, the majority of RSV cases were observed between calendar week 41 of 2017 (October) and week 22 of 2018 (May). Two additional cases were observed in calendar weeks 28 (July) and 31 (August) of 2018. In the 2018/2019 season, most cases were observed between the weeks 47 of 2018 (November) and 22 of 2019 (May). In addition, a single case was detected in week 30 (July). In the season of 2019/2020, the cases were mainly observed between week 45 of 2019 (November) and week 14 of 2020 (April). An additional case was detected in June 2020 (week 24). During all three pre-pandemic seasons, the peak of RSV detections occurred in February (week 6 of 2018 and 2019, and week 7 in 2020). In the 2021/2022 season, the cases were observed between week 27 of 2021 (June) and week 9 of 2022 (February). Peak RSV detections occurred in calendar week 40 of 2021 (September/October).

A case detected in a returning traveler from Africa and a case detected in a child in December 2020 and March 2021, respectively, were excluded from further analysis because they represent the only two cases of season 2020/2021. Five RSV detections after March 2022 were considered to represent cases of a new season (2022/2023) and not analyzed further.

**Figure 2:**
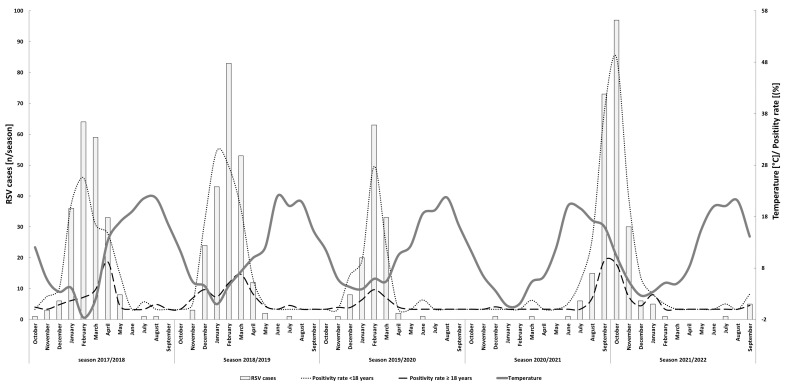
Monthly RSV cases (*n* = 803), positivity rates of the RSV assay and mean temperature during the study period. Note the two different y-axes: the left axis refers to the absolute numbers of detected RSV cases (light grey bars) while the right axis refers to the mean temperature of the respective month [°C] (dark grey line), the positivity rate for the pediatric patients under the age of 18 years [%] (black dotted line), and the positivity rates for adult patients [%] (black dashed line). The x-axis is labeled according to the pre-pandemic definition that was used to define the start and end of a respiratory season.

Except for season 2021/2022, peak RSV detections coincided with the lowest temperatures of the year (Figure 2). In season 2017/2018, peak RSV detections for adult cases were observed in March while peak pediatric cases were observed in February. Peak RSV detections occurred at the same time for the adult and pediatric patients in each of the other seasons of the study period (Supplementary Figure S1). The overall RSV PCR positivity rates for the main detection intervals mentioned above ranged from 4.0% (143/3562) in the season of 2019/2020 to 11.5% (252/2196) in the season of 2021/2022, and were 6.4% (248/3889) and 8.3% (253/3039) in the seasons of 2017/2018 and 2018/2019, respectively.

However, marked differences were observed when the positivity rates for pediatric and adult samples were examined independently (Figure 2). In the adult cohort, the peak positivity rates for seasons 2017/2018, 2018/2019, 2019/2020, and 2021/2022 were 3.8%, 6.8%, 3.8%, and 9.4%, respectively. For the pediatric patients, the peak positivity rates were 25.6%, 30.7%, 27.7%, and 48.9%, respectively.

**Figure 3:**
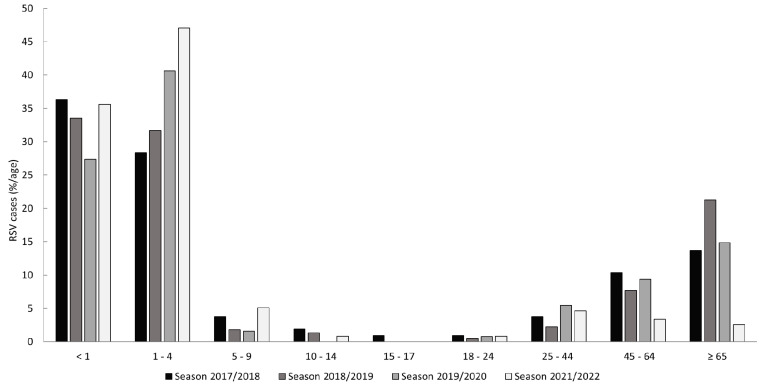
Relative distribution of RSV cases (*n* = 796) by age. The bars represent the relative amount of cases detected in the indicated age group for each of the analyzed seasons.

A shift between dominant RSV subtypes was observed during the study period (Table 1). While the majority of cases originated from infections with RSV-B in season 2017/2018, RSV-A infections were predominantly observed thereafter. Compared to the three pre-pandemic seasons, significantly more pediatric cases were observed in season 2021/2022 (Table 1, *p* < 0.001 in comparison to all other seasons). The relative age stratification of RSV cases by season is shown in Figure 3.

### 3.2. Phylogenetic Analysis

The *G gene* amplification approach was applied to the samples of all cases of the season 2021/2022 and to randomly selected samples of cases from the three pre-pandemic seasons for both RSV-A and RSV-B. For RSV-A, the genotyping approach was successful for 130 (95.6%) cases in season 2021/2022. Additionally, genotyping was performed for 15 cases of season 2017/2018, 19 cases of season 2018/2019, and 17 cases of season 2019/2020. An infection with two different strains, derived from minor variants in the genotyping approach, was observed in one case in season 2018/2019 (RSVA/Leipzig/2019/7) and two cases in season 2021/2022 (RSVA/Leipzig/2021/39 and RSV/Leipzig/2021/64). For RSV-B, the genotyping approach was successful for 96 (92.3%) cases in season 2021/2022. Additionally, genotyping was performed for 15 cases of season 2017/2018, 15 cases of season 2018/2019, and 18 cases of season 2019/2020. Within the genotyped subset, respiratory specimens included 296 nasal and/or oropharyngeal swabs (91.1%), 12 throat rinsing fluids (3.7%), eight broncho-alveolar lavage fluids (2.5%), six nasal secretions (1.8%), and three tracheal secretions (0.9%).

**Figure 4:**
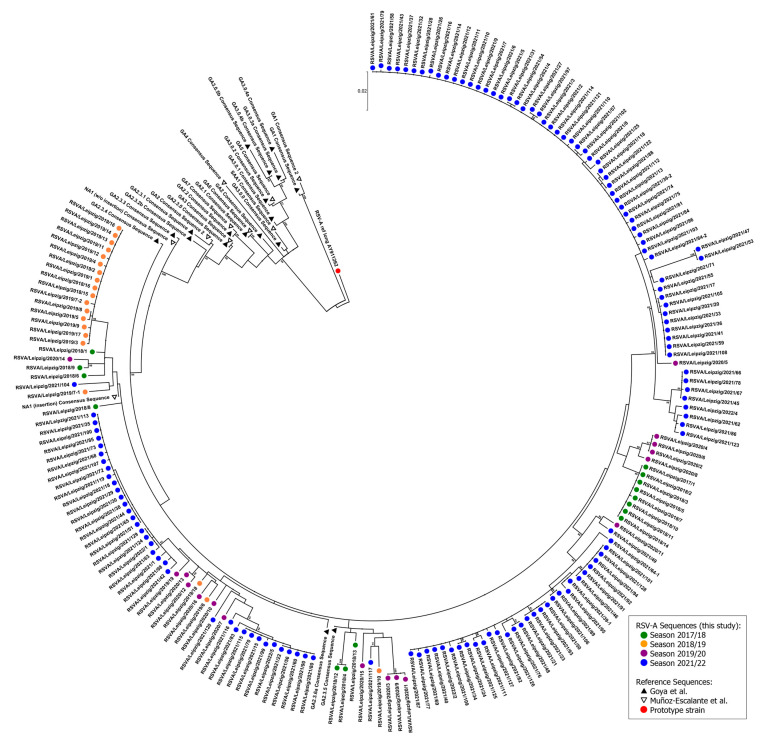
Molecular phylogenetic analysis of the RSV-A *G gene* by maximum likelihood method. The evolutionary history was inferred by using the maximum likelihood method based on the Tamura–Nei model [24]. The tree with the highest log likelihood (−6313.50) is shown. Initial tree(s) for the heuristic search were obtained automatically by applying neighbor-joining and BioNJ algorithms to a matrix of pairwise distances estimated using the maximum composite likelihood (MCL) approach, and then selecting the topology with superior log likelihood value. The tree is drawn to scale, with branch lengths measured in the number of substitutions per site. The analysis involved 214 nucleotide sequences. There were a total of 966 positions in the final dataset. Evolutionary analyses were conducted in MEGA7 [23]. Only nodes with a statistical support >80% are shown. The symbols indicate the sequence origin or the season of the indicated strain (dots: red, RSV-A reference strain; green: season 2017/2018 isolates; orange, season 2018/2019 isolates; purple, season 2019/2020 isolates; blue, season 2021/2022 isolates; black triangle: consensus reference sequences according to Goya et al. [14]; white triangle, consensus reference sequences according to Muñoz-Escalante et al. [16]).

Except for three isolates, the coding sequences (CDS) of the RSV-A *G gene* encompassed 966 bases translating into 321 amino acids. The CDS had a length of 969 bases for two isolates (RSVA/Leipzig/2020/15 of season 2019/2020 and RSVA/Leipzig/2019/7-1 of season 2018/2019) while one isolate showed a truncated CDS of 933 bases (RSVA/Leipzig/2020/11 of season 2019/2020). The phylogenetic analysis of the complete *G gene* revealed a close relationship between all RSV-A isolates analyzed (Figure 4). The nucleotide identity was 97.5% for the isolates of season 2021/2022 and 97.2% for all analyzed sequences. No distinct well-supported cluster was observed for any of the four seasons as most groupings of different isolates are located in terminal nodes with low statistical support of ancestral nodes. The isolates clustered together with the consensus sequences of GA2.3.5 (Goya et al. [14]) and NA1 (Muñoz-Escalante et al. [16]), which was also confirmed by separate phylogenetic analyses carried out with proposed reference strains (Supplementary Figures S2 and S3).

**Figure 5:**
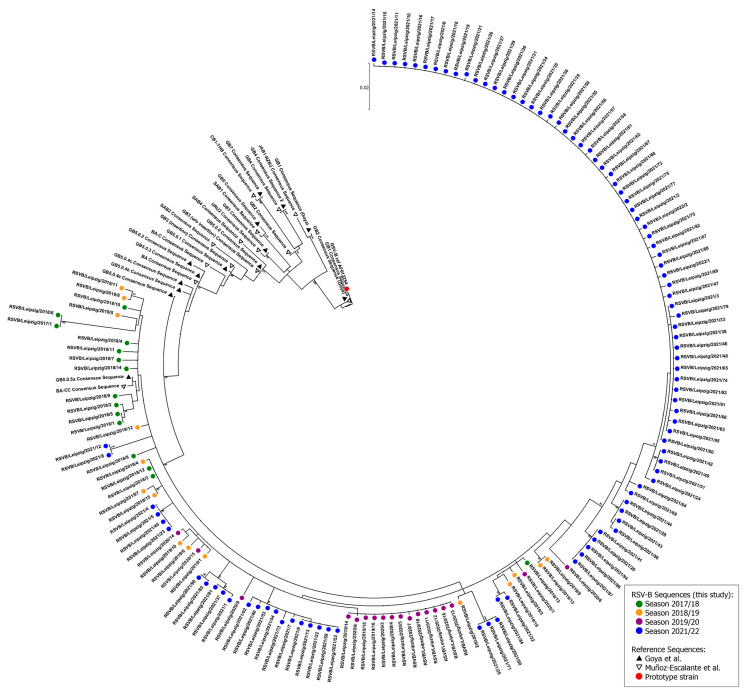
Molecular phylogenetic analysis of the RSV-B *G gene* by maximum likelihood method. The evolutionary history was inferred by using the maximum likelihood method based on the Tamura–Nei model [24]. The tree with the highest log likelihood (−4492.48) is shown. Initial tree(s) for the heuristic search were obtained automatically by applying neighbor-joining and BioNJ algorithms to a matrix of pairwise distances estimated using the maximum composite likelihood (MCL) approach, and then selecting the topology with superior log likelihood value. The tree is drawn to scale, with branch lengths measured in the number of substitutions per site. The analysis involved 173 nucleotide sequences. There were a total of 939 positions in the final dataset. Evolutionary analyses were conducted in MEGA7 [23]. Only nodes with a statistical support >80% are shown. The symbols indicate the sequence origin or the season of the indicated strain (dots: red, RSV-B reference strain; green: season 2017/2018 isolates; orange, season 2018/2019 isolates; purple, season 2019/2020 isolates; blue, season 2021/2022 isolates; black triangle: consensus reference sequences according to Goya et al. [14]; white triangle, consensus reference sequences according to Muñoz-Escalante et al. [15]).

The CDS length of the majority of the RSV-B *G genes* encompassed 933 bases translating into 310 amino acids. The CDS had a length of 954 bases in nine isolates (including isolates of all seasons) while two isolates showed a CDS of 942 bases (RSVB/Leipzig/2021/85 and RSVB/Leipzig/2021/90 of season 2021/2022). The phylogenetic analysis of the complete *G gene* revealed a close relationship between all analyzed RSV-B isolates (Figure 5). The nucleotide identity was 98.5% for the isolates of season 2021/2022 and 98.3% for all analyzed sequences. Again, no distinct well-supported cluster was observed for any of the four seasons as most groupings of different isolates are located in terminal nodes with low statistical support of ancestral nodes. The isolates clustered together with the consensus sequences of GB5.0.5a (Goya et al. [14]) and BA-CC (Muñoz-Escalante et al. [15]), which was also confirmed in the separate phylogenetic analyses with proposed reference strains (Supplementary Figures S4 and S5).

### 3.3. Study Population and Clinical Features

The RSV-related patient characteristics and clinical parameters are presented in Table 2 for the pediatric cohort and in Table 3 for the adult cohort. A comparison between the pre-pandemic seasons of 2017/2018, 2018/2019, and 2019/2020 and the season of 2021/2022 was performed.

In the pediatric cohort, neither the median age nor the gender differed between the investigated seasons. However, a higher percentage of siblings was noted for the season of 2021/2022 (*p* = 0.004) as well as a higher frequency of outpatients (*p* = 0.022). No differences were observed in comorbidities and risk factors for a severe disease course. RSV infections in the season of 2021/2022 were associated with a lower frequency of fever (*p* = 0.007), pneumonia (*p* = 0.038), and the usage of budesonide inhalations (*p* = 0.016). Furthermore, the amount of co-infections was higher in the pre-pandemic seasons (*p* = 0.001) and showed a different composition of pathogen types (*p* = 0.006). In the corresponding post hoc pairwise analysis, the main difference was found in the presence of bacterial co-pathogens (*p* = 0.029), which was lower in the season of 2021/2022. The detected co-pathogens are listed in Supplementary Table S12.

For the adult cohort, the mean age of the patients was significantly younger in season 2021/2022 when compared to the pre-pandemic seasons (47.07 versus 64.7 years; *p* < 0.001). Additionally, arterial hypertension (*p* = 0.048) and other cardiovascular diseases (*p* = 0.023) were less frequently observed in the season of 2021/2022. No other significant differences for comorbidities and risk factors for a severe disease course, or clinical outcome parameters such as LRTI, intensive care unit (ICU) admission, or in-hospital mortality, were observed. The detected co-pathogens are listed in Supplementary Table S13.

## 4. Discussion

In the wake of the SARS-CoV-2 pandemic, NPIs [17,25] were introduced in Germany in March 2020 and were regulated on both national and regional levels [26,27,28,29]. Specifically, the NPIs involved several nationwide lockdowns between March and April 2020 and between December 2020 and March 2021 including the closing of schools and daycare centers. Although still in effect in 2023, NPIs were alleviated starting in May 2021 [30] and tied to the incidence of SARS-CoV-2 infections. In addition to legal regulations, the pandemic had a tremendous impact of on societal behaviors, such as mask wearing in public and increased work-from-home practices [31]. The aim of this study was to describe the local seasonal and molecular epidemiology and clinical characteristics observed during the resurgence of RSV infections in the summer and autumn of 2021.

Although different methodical approaches for the definition of a season exist, RSV seasons in Europe typically start in December and last until April of the following year [32,33,34]. For Germany, the Robert Koch Institute (RKI) recently published a new proposal for the definition of the start and endpoints of the RSV season to more accurately correspond to the endemic circulation of RSV [35]. Acknowledging the high burden of RSV infections and clinical importance of this subgroup, a season was defined based on the positivity rate (PR) of RSV real-time RT-PCR detection assays in children between 0 and 4 years of age as assessed by the national outpatient sentinel surveillance. In particular, an RSV epidemic season was defined as starting with the first of two consecutive weeks in which the lower limit of a 95% confidence interval of the PR exceeds 5% and ends by the week that precedes the first of two consecutive weeks in which the lower limit drops below 5%. Correspondingly, the epidemic circulation for RSV for the pre-pandemic seasons spanned between week 51 (December) to week 13 (March) for season 2017/2018, week 50 (December) to week 11 (March) for season 2018/2019, and week 51 (December) to week 12 (March) for season 2019/2020. Seasonal peaks were reported for week four, week three (both in January), and week six (February), respectively. With the use of PRs, a season is confined to a highly prevalent circulation of RSV acknowledging possible earlier and later detections. The pre-pandemic seasons of the current study are in line with these proposed seasons, as the detection intervals were framed with months of low PRs. Furthermore, the high burden of RSV infections in young children under the age of four is confirmed within an inpatient population (Figure 2 and Figure 3). Of note, although the interval for the epidemic circulation of season 2019/2020 resembles seasons 2017/2018 and 2018/2019, data from the present study including all age groups suggest an earlier end of RSV circulation in 2020. Likely, this is also connected to the beginning of contact restriction measures and behavioral changes in the wake of the then emerging SARS-CoV-2 pandemic. Nevertheless, the strongest impact of the contact restrictions was observed for seasons 2020/2021, in which only two RSV cases could be confirmed, and the season of 2021/2022. According to the RKI [35], endemic circulation started in week 35 (August) and ended in week 50 (December) of 2021, peaking 19 weeks before the usual average (week eight) in week 41 (October), which is in line with the current study.

Similar effects of social distancing policies and behavioral changes were also observed for other non-SARS-CoV-2 respiratory pathogens [31,36]. In temperate climates, strictly seasonal or cyclic circulation patterns are a common feature of numerous viral infections such as non-polio enteroviruses [37,38], influenza viruses [39,40], and parainfluenza viruses [41]. Instead of an expected peak in 2020, an upsurge of enterovirus D68 was observed in autumn of 2021 across Europe. In addition to RSV [42,43,44], interseasonal peaks were also reported for parainfluenza viruses [45,46]. It must be stressed that the resurgence of common respiratory pathogens is highly heterologous and the disruption of the usual circulation patterns may have had a tremendous and yet unprecedented impact on the genetic diversity of respiratory viruses. This became evident by the heavily suppressed circulation of influenza viruses in 2020 and 2021 with a reduced genetic diversity and the absence of the Yamagata lineage of influenza B [47]. Thus, continued phylodynamic studies are urgently needed.

Despite the clinical and epidemiological importance, the surveillance of the genetic diversity of RSV is hampered by the lack of a global surveillance system comparable to influenza virus or, since its emergence, SARS-CoV-2. Furthermore, there is no standardized or internationally recognized and curated classification system for the establishment of unified RSV genotyping criteria [12,48,49,50,51,52]. Recently, two independent groups published proposals for a reclassification of both RSV-A and RSV-B genotypes based on the ectodomain of the *G gene* [14,15,16]. However, due to methodical differences, e.g., the cut-off for an intragenotype p-distance (0.03 for RSV-A and RSV-B [14] vs. 0.037 for RSV A and 0.0358 for RSV-B [15,16]), there is still no consensus about the amount of genotypes per RSV subtype, as well as of subgenotypes or lineages. The circulation pattern and presence of different RSV genotypes showed transitions through time [14,15,16]. Publicly available sequence data suggest that numerous proposed genotypes have gone extinct and that after a period of diversification in the last century, a global predominance of single genotypes in RSV-A and B has once again evolved. In particular, these have been genotypes termed “ON1” for RSV-A, after the initial identification in strains collected in the season of 2010/2011 in Ontario, Canada [53], and “BA” for RSV-B, after the initial identification in strains collected in 1999 in Buenos Aires, Argentina [54]. Intriguingly, both genotypes show a partial duplication (72 nucleotides for RSV-A and 60 nucleotides for RSV-B) in the 2nd hypervariable region of the *G gene*. In accordance with these observations, all isolates of the current study belonged to the same genotype, regardless of whether they originated from cases before or after NPIs in the wake of the SARS-CoV-2 pandemic. In particular, these are genotypes NA1 [16] or GA2.3.5 [14] (corresponding to ON1) for RSV-A and BA-CC [15] or GB5.0.5a [14] (corresponding to BA) for RSV-B. The contact restriction measures might have constituted an evolutionary bottleneck, accelerating the extinction of less common genotypes or even different lineages of the same genotype [42].

Furthermore, this observation highlights the importance of the pre-existing immunity and its impact on RSV circulation and possibly even seasonality (see above), as the reemergence of RSV in 2021 apparently was not caused by major antigenic shifts circumventing a pre-existing immune response. More likely, it seems that an immunologic gap caused by the lack of RSV circulation during the period of intensified contact restrictions was created within the population [55]. A similar phenomenon was hypothesized for the severe influenza virus season of 2017/2018. The Yamagata lineage of influenza B was not covered by the then commonly used (trivalent) vaccine compositions and only circulated on a low level in the two previous seasons [39]. Thus, the low pre-existing immunity in the population might have contributed to the high case numbers observed in the season of 2017/2018. Furthermore, the cyclic patterns of different enterovirus serotypes could be predicted by integrating population-based immunity models [56,57]. For RSV, the importance of a waning immunity was already shown in a prospective study using samples from the Danish National Birth Cohort. An inverse correlation of RSV-related hospitalization in children under six months of age and the levels of neutralizing antibodies in cord blood samples was observed in temporal association to RSV seasons [58].

The short-lived or transient immunity may have been a major contributor of the observed resurgence of RSV cases and thus could have had a direct influence on the epidemiologic and clinical characteristics of the observed cases. There was a remarkable age disparity in the circulation of RSV in the resurgence of 2021/2022, as evidenced by the significantly higher percentage of children when compared to the pre-pandemic seasons (Table 1). Similar observations were also reported in other studies. Furthermore, a shift towards older age for children and younger age for adults was observed [59,60,61]. In addition to immunologic factors, this shift may also be reflective of a higher adhesion to NPIs in the adult population. In the presented pediatric cohort, age was not significantly different from the pre-pandemic mean. However, since children under the age of two years have the highest risk for hospitalization [62], older children may be underrepresented in the current study. Nevertheless, significantly more children with siblings in the household were noted for 2021/2022. For the adult cohort the mean age of the patients in 2021/2022 was significantly younger as compared to the pre-pandemic mean (64.7 vs. 47.07 years, *p* < 0.001), which is in line with observations by Falsey et al. [59].

It is difficult to assess whether these shifts in the patient’s age were caused by the need for pathogen identification due to clinical symptoms or a more liberal utilization of respiratory pathogen tests, including multiplex tests, due to the SARS-CoV-2 pandemic. It is therefore noteworthy that for both the pediatric and the adult cohort the assessed clinical characteristics were largely similar to what was observed before the SARS-CoV-2 pandemic.

In agreement with other studies [5,6,7], the incidence of comorbidities that may contribute to a severe disease course was generally high in the adult cohort. Except for immunosuppression, there was a trend towards a lower incidence of comorbidities in the season 2021/2022, with significant differences for the parameters arterial hypertension and cardiovascular diseases. Reasons for this observation might be the younger mean age of the adults or a slightly differential comorbidity profile that is associated with a reduced risk of hospitalization due to cardiovascular risk factors at higher ambient temperatures [63,64]. Nevertheless, the overall high amount of chronic kidney failure, cardiac insufficiency, malignancy and immunosuppression was also observed in patients infected with other respiratory pathogens at the same hospital, including influenza B virus [39], parainfluenza virus type 3 [41], and rhinovirus [19]. The especially high amount of ICU admissions (25%) and need for oxygen supply (23.3%) of the studied RSV cases are comparable to what was observed for influenza virus (20–25.8% and 12.6–17.1%, respectively) [39] and highlights the susceptibility of these patients to develop a severe RSV infection.

For the pediatric cohort, most of the analyzed clinical parameters did not differ in comparison to the pre-pandemic seasons and comorbidities were especially rare. The observation of high rates of LRTI (82.4%), including cases with bronchiolitis (67.1%) and pneumonia (19%), as well as the need for intensive care treatment (10.5%), are in line with other reports in Germany. These findings underline the high utilization of hospital resources to treat RSV infections [65,66]. An increased [44] or slightly reduced [43] severity of RSV cases was reported after the alleviation of NPIs, which was most likely dependent on the amount of cases that were enrolled through an emergency department. The notion of an increased amount of outpatients, fewer cases with fever and pneumonia, and a reduced use of budesonide inhalations in the current study, however, indicate similarities to observations made in another tertiary care center in Western Australia [43]. Interestingly, differences were noted for the overall rate of co-infections and of the pathogen type. Fewer co-infections were noted for season 2021/2022 (25.9%) in comparison to the pre-pandemic seasons (38.7%, *p* = 0.001). A statistically significant difference could be observed for the (lesser) amount of bacterial co-infections. In Germany, an upsurge of invasive pneumococcal disease was observed in spring and summer of 2021, with an increased dynamic in children below four years of age [67]. The high variability that was observed for the resurgence of viral respiratory pathogens (see above) can also likely be assumed for bacterial pathogens, and possibly contributes to the altered profile of detected co-infections. With regard to other respiratory pathogens studied at the same hospital, there is a remarkable resemblance of the incidence of fever (36.4%), bronchiolitis/bronchitis (67.1%), and the administration of bronchodilators (73%) with rhinovirus species C infections (32.4%, 57.4%, and 73.6%, respectively). These findings support a growing number of studies underlining the importance of RSV and rhinoviruses in the association with severe bronchiolitis and the development of wheezing and asthma [4,68,69].

Several limitations of this study should be noted. Notably, the genotyping approach was not successful for all RSV cases and may be a source of bias. A possible explanation might be the higher sensitivity of the nucleic acid amplification test used for the RSV detection. However, the authors are confident that the successful genotyping of more than 95% of RSV-A and more than 92% of RSV-B isolates constitute an adequate representation of the local genotype circulation of the season 2021/2022. Due to the analysis of only a fraction of RSV isolates before the SARS-CoV-2 pandemic, however, the circulation of other less common genotypes cannot be ruled out. Asthma was probably underestimated in the pediatric cohort due to the need for lung function tests for an appropriate diagnosis, which cannot be undertaken with patients under the age of five. Due to the retrospective study design, only associations could be shown, without proof of causality. Patient selection favoring severe cases may have occurred due to the sampling at a tertiary care hospital, which is underlined by the high numbers of pneumonia cases and cases that needed an ICU stay. In addition, the age spectrum of the analyzed patients is likely to underrepresent patients who do not belong to the high-risk groups for severe disease courses and hospitalization. Detection of a further pathogen was considered as a co-infection; however, especially for bacterial pathogens, colonization cannot be ruled out. Finally, due to the limited number of adult RSV cases in the season of 2021/2022, this study lacks the power to determine potential further differences in comparison to the pre-pandemic RSV cases in that age group.

## 5. Conclusions

This study reports on the epidemiology and associated clinical spectrum of RSV cases that were treated at a tertiary care university hospital in Germany before and after the implementation of non-pharmaceutical interventions in the wake of the SARS-CoV-2 pandemic. The findings are consistent with other studies and indicate a profound impact of pre-existing immunity and adherence to contact restrictions measures on the resurgence of RSV in pediatric and adult populations. Furthermore, the close phylogenetic relatedness of circulating strains is evidenced by analysis of the *G gene*. Additional epidemiologic studies or population-based surveillance programs are warranted, as behavioral changes are likely to have an influence on the circulation of respiratory pathogens for the near future. Moreover, the probable implementation of a recently approved monoclonal antibody with an extended half-life in clinical treatment guidelines [11,70] as well as potential future vaccination strategies [71], may necessitate an analysis of the genetic sequence of the fusion protein going forward.

## Figures and Tables

**Table 1:** Gender, age, and RSV species distribution during the study period.

		Season 2017/2018	Season 2018/2019	Season 2019/2020	Season 2021/2022	Total		*p*-Value
age group								
<18	[% (*n*/total)]	71.4 (152/213)	68.3 (151/221)	69.5 (89/128)	88.9 (208/234)	75.4 (600/796)	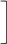	<0.001
≥18	[% (*n*/total)]	28.6 (61/213)	31.7 (70/221)	30.5 (39/128)	11.1 (26/234)	24.6 (196/796)		
gender								
male	[% (*n*/total)]	62.9 (134/213)	50.7 (112/221)	62.5 (80/128)	54.7 (128/234)	57.0 (454/796)	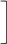	n.s.
female	[% (*n*/total)]	37.1 (79/213)	49.3 (109/221)	37.5 (48/128)	45.3 (106/234)	43.0 (342/796)		
RV species								
RSV-A	[% (*n*/total)]	24.9 (53/213)	59.7 (132/221)	77.3 (99/128)	56.0 (131/234)	52.1 (415/796)	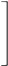	<0.001
RSV-B	[% (*n*/total)]	75.1 (160/213)	38.9 (86/221)	21.9 (28/128)	41.9 (98/234)	46.9 (373/796)		
mixed	[% (*n*/total)]	0 (0/213)	1.4 (3/221)	0.8 (1/128)	2.1 (5/234)	1.1 (9/796)		

Analyzed categories are displayed on the column to the left and given as relative and absolute frequencies [% (n/total)]. (*n*/total) indicates the respective cases of the total amount of cases of the respective season. The *p*-values of the Chi-square tests for the contingency tables including all seasons and subgroups are indicated. Significant *p*-values for the post hoc pairwise analysis of specific categories are given in the corresponding paragraph. n.s., not significant.

**Table 2:** Study population and clinical features of pediatric RSV cases.

		Pre-Pandemic	2021/2022	Total		*p*-Value
**study population**						
female	[% (*n*/total)]	41.6 (163/392)	45.6 (95/208)	43.0 (258/600)	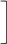	n.s.
male	[% (*n*/total)]	58.4 (229/392)	54.3 (113/208)	57.0 (342/600)		
age [years]	[mean ± SD]	1.2 ± 2.3	1.4 ± 1.98	1.3 ± 2.20		n.s.
gestational week at birth	[mean ± SD]	38.1 ± 3.64	38 ± 3.68	38.1 ± 3.66		n.s.
preterm birth	[% (*n*/total)]	19.2 (61/318)	20.3 (36/177)	19.6 (97/495)		n.s.
siblings	[% (*n*/total)]	62.1 (231/372)	74.1 (143/193)	66.2 (374/565)		**0.004**
twins	[% (*n*/total)]	4.6 (17/373)	3.6 (7/193)	4.2 (24/566)		n.s.
caesarean section	[% (*n*/total)]	16.0 (60/375)	17.0 (32/188)	16.3 (92/563)		n.s.
RSV prophylaxis	[% (*n*/total)]	3.1 (12/392)	3.4 (7/208)	3.2 (19/600)		n.s.
inpatients	[% (*n*/total)]	97.2 (381/392)	93.3 (194/208)	95.8 (575/600)	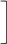	**0.022**
outpatients	[% (*n*/total)]	2.8 (11/392)	6.7 (14/208)	4.2 (25/600)		
length of hospital stay [days]	[mean ± SD]	6.6 ± 7.99	6.1 ± 8.19	6.5 ± 8.06		n.s.
**comorbidities and risk factors**						
asthma	[% (*n*/total)]	1.0 (4/392)	1.0 (2/207)	1.5 (9/599)		n.s.
COPD	[% (*n*/total)]	0 (0/392)	0 (0/207)	0 (0/599)		n.s.
lung transplant	[% (*n*/total)]	0 (0/392)	0 (0/207)	0 (0/599)		n.s.
chronic kidney failure	[% (*n*/total)]	0.3 (1/392)	0 (0/207)	0.2 (1/599)		n.s.
cardiac failure	[% (*n*/total)]	1.8 (7/392)	1.9 (4/207)	1.8 (11/599)		n.s.
arterial hypertension	[% (*n*/total)]	0.5 (2/392)	1.0 (2/207)	0.7 (4/599)		n.s.
cardiovascular diseases	[% (*n*/total)]	0.5 (2/392)	1.4 (3/207)	0.8 (5/599)		n.s.
diabetes	[% (*n*/total)]	0.8 (3/392)	0.5 (1/207)	0.7 (4/599)		n.s.
malignancy	[% (*n*/total)]	2.0 (8/392)	1.4 (3/207)	1.8 (11/599)		n.s.
immunosuppression	[% (*n*/total)]	1.8 (7/392)	2.4 (5/207)	2.0 (12/599)		n.s.
**clinical presentation and features**						
nosocomial	[% (*n*/total)]	0.2 (1/391)	0.4 (1/207)	0.3 (2/598)		n.s.
symptomatic	[% (*n*/total)]	97.4 (380/390)	94.2 (195/207)	96.3 (575/597)		**0.046**
fever	[% (*n*/total)]	40.3 (157/389)	29.1 (60/206)	36.4 (217/595)		**0.007**
URTI	[% (*n*/total)]	74.4 (268/360)	76.0 (140/184)	75.0 (408/544)		n.s.
pharyngitis	[% (*n*/total)]	68.2 (237/347)	72.1 (127/176)	69.5 (364/523)		n.s.
LRTI	[% (*n*/total)]	82.0 (321/391)	83.0 (171/206)	82.4 (492/597)		n.s.
bronchiolitis/bronchitis	[% (*n*/total)]	65.2 (255/391)	70.8 (146/206)	67.1 (401/597)		n.s.
pneumonia	[% (*n*/total)]	21.4 (84/391)	14.4 (30/207)	19.0 (114/598)		**0.038**
respiratory failure	[% (*n*/total)]	57.2 (224/391)	61.1 (126/206)	58.6 (350/597)		n.s.
ICU stay	[% (*n*/total)]	12.0 (47/391)	7.6 (16/208)	10.5 (63/599)		n.s.
length of ICU stay [days]	[mean ± SD]	9.3 ± 11.14	7.4 ± 6.39	8.8 ± 10.06		n.s.
assisted ventilation	[% (*n*/total)]	2.0 (8/390)	1.4 (3/208)	1.8 (11/599)		n.s.
oxygen supply	[% (*n*/total)]	59.7 (233/390)	57.9 (120/207)	59.1 (353/597)		n.s.
inhalation	[% (*n*/total)]	84.1 (328/390)	80.6 (167/207)	82.9 (495/597)		n.s.
adrenalin	[% (*n*/total)]	7.2 (28/385)	5.5 (11/200)	6.6 (39/585)		n.s.
salbutamol	[% (*n*/total)]	70.5 (275/390)	64.7 (134/207)	69.9 (409/585)		n.s.
ipratropium bromide bromide	[% (*n*/total)]	17.6 (68/385)	17.5 (35/200)	17.6 (103/585)		n.s.
budesonide	[% (*n*/total)]	15.5 (60/385)	8.5 (17/200)	13.1 (77/585)		**0.016**
methylxanthine administration	[% (*n*/total)]	0.7 (3/390)	0.4 (1/207)	0.6 (4/597)		n.s.
syst. prednisolone administration	[% (*n*/total)]	34.8 (136/390)	31.8 (66/207)	33.8 (202/597)		n.s.
co-infections	[% (*n*/total)]	38.7 (152/392)	25.9 (54/208)	34.3 (206/600)		**0.001**
bacterial	[% (*n*/total)]	9.9 (39/392)	4.8 (10/208)	8.1 (49/600)	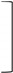	**0.006**
viral	[% (*n*/total)]	25.0 (98/392)	19.2 (40/208)	23.0 (138/600)		
fungal	[% (*n*/total)]	0 (0/392)	0.4 (1/208)	0.1 (1/600)		
combined	[% (*n*/total)]	3.8 (15/392)	1.4 (3/208)	3.0 (18/600)		
in-hospital mortality	[% (*n*/total)]	0 (0/392)	0 (0/208)	0 (0/600)		n.s.

Analyzed categories are displayed in the column to the left and are either given as frequencies (%), range [median(range)], or as means and standard deviations (mean ± SD). (*n*/total) indicates the respective cases for the total amount of available data. The *p*-values of the Chi-square tests for the contingency tables including all subcategories are indicated. All significant *p*-values for the post hoc pairwise analysis are given in the corresponding paragraph. The Mann–Whitney U test was performed to compare continuous variables. COPD, chronic obstructive pulmonary disease; URTI, upper respiratory tract infection; LRTI, lower respiratory tract infection; ICU, intensive care unit; n.s., not significant; syst., systemic.

**Table 3:** Study population and clinical features of adult RSV cases.

		Pre-Pandemic	2021/2022	Total		*p*-Value
**study population**						
female	[% (*n*/total)]	42.9 (73/170)	42.3 (11/26)	42.8 (84/196)	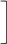	n.s.
male	[% (*n*/total)]	57.1 (97/170)	57.6 (15/26)	57.1 (112/196)		
age [years]	[mean ± SD]	64.7 ± 16.98	47.07 ± 17.82	62.4 ± 18.10		**<0.001**
inpatients	[% (*n*/total)]	79.4 (135/170)	76.0 (19/25)	78.9 (154/195)	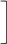	n.s.
outpatients	[% (*n*/total)]	20.6 (35/170)	24.0 (6/25)	21.0 (41/195)		
length of hospital stay [days]	[mean ± SD]	18.5 ± 15.62	17 ± 22.71	18.3 ± 16.54		n.s.
**comorbidities and risk factors**						
asthma	[% (*n*/total)]	5.3 (9/168)	8.3 (2/24)	5.7 (11/192)		n.s.
COPD	[% (*n*/total)]	17.9 (30/168)	12.5 (3/24)	17.1 (33/192)		n.s.
lung transplant	[% (*n*/total)]	4.7 (8/170)	3.8 (1/26)	4.5 (9/196)		n.s.
chronic kidney failure	[% (*n*/total)]	39.9 (67/168)	20.8 (5/24)	37.5 (72/192)		n.s.
cardiac failure	[% (*n*/total)]	19.0 (32/168)	8.3 (2/24)	17.7 (34/192)		n.s.
arterial hypertension	[% (*n*/total)]	58.9 (99/168)	37.5 (9/24)	56.2 (108/192)		**0.048**
cardiovascular diseases	[% (*n*/total)]	61.9 (104/168)	37.5 (9/24)	58.8 (113/192)		**0.023**
diabetes	[% (*n*/total)]	31.5 (53/168)	29.1 (7/24)	31.2 (60/192)		n.s.
malignancy	[% (*n*/total)]	35.7 (60/168)	29.1 (7/24)	34.8 (67/192)		n.s.
immunosuppression	[% (*n*/total)]	36.3 (61/168)	50.0 (12/24)	38.0 (73/192)		n.s.
**clinical presentation and features**						
nosocomial	[% (*n*/total)]	4.7 (8/169)	12.0 (3/25)	5.6 (11/194)		n.s.
symptomatic	[% (*n*/total)]	78.7 (126/160)	85.7 (18/21)	79.5 (144/181)		n.s.
fever	[% (*n*/total)]	24.0 (38/158)	21.0 (4/19)	23.7 (42/177)		n.s.
URTI	[% (*n*/total)]	35.4 (28/79)	33.3 (4/12)	35.1 (32/91)		n.s.
pharyngitis	[% (*n*/total)]	11.2 (7/62)	27.2 (3/11)	13.6 (10/73)		n.s.
LRTI	[% (*n*/total)]	49.3 (78/158)	63.1 (12/19)	50.8 (90/177)		n.s.
bronchitis	[% (*n*/total)]	7.5 (12/158)	15.7 (3/19)	8.4 (15/177)		n.s.
pneumonia	[% (*n*/total)]	33.3 (53/159)	42.1 (8/19)	34.2 (61/178)		n.s.
respiratory failure	[% (*n*/total)]	28.9 (46/159)	36.8 (7/19)	29.7 (53/178)		n.s.
ICU stay	[% (*n*/total)]	25.8 (44/170)	19.2 (5/26)	25 (49/196)		n.s.
length of ICU stay [days]	[mean ± SD]	8.2 ± 11.91	3.5 ± 3.72	7.6 ± 11.3		n.s.
assisted ventilation	[% (*n*/total)]	8.2 (14/170)	7.6 (2/26)	8.1 (16/196)		n.s.
oxygen supply	[% (*n*/total)]	23.8 (40/168)	20.0 (5/25)	23.3 (45/193)		n.s.
inhalation	[% (*n*/total)]	18.4 (31/168)	16.6 (4/24)	18.2 (35/192)		n.s.
adrenalin	[% (*n*/total)]	0 (0/166)	0 (0/24)	0 (0/190)		n.s.
salbutamol	[% (*n*/total)]	10.7 (18/168)	4.1 (1/24)	10.0 (19/190)		n.s.
ipratropium bromide bromide	[% (*n*/total)]	9.0 (15/166)	4.1 (1/24)	8.3 (16/190)		n.s.
budesonide	[% (*n*/total)]	0.6 (1/166)	4.1 (1/24)	1.0 (2/190)		n.s.
syst. prednisolone administration	[% (*n*/total)]	14.8 (25/168)	12.5 (3/24)	14.5 (28/192)		n.s.
co-infection	[% (*n*/total)]	21.1 (36/170)	19.2 (5/26)	20.9 (41/196)		n.s.
bacterial	[% (*n*/total)]	5.8 (10/170)	7.6 (2/26)	6.1 (12/196)	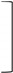	n.s.
viral	[% (*n*/total)]	11.1 (19/170)	11.5 (3/26)	11.2 (22/196)		
fungal	[% (*n*/total)]	1.1 (2/170)	0 (0/26)	1.0 (2/196)		
combined	[% (*n*/total)]	2.9 (5/170)	0 (0/26)	2.5 (5/196)		
in-hospital mortality	[% (*n*/total)]	6.4 (11/170)	3.8 (1/26)	6.1 (12/196)		n.s.

Analyzed categories are displayed in the column to the left and are either given as frequencies (%), range [median(range)], or as means and standard deviations (mean ± SD). (*n*/total) indicates the respective cases for the total amount of available data. The *p*-values of the Chi-square tests for the contingency tables including all subcategories are indicated. All significant *p*-values for the post hoc pairwise analysis are given in the corresponding paragraph. The Mann–Whitney U test was performed to compare continuous variables. COPD, chronic obstructive pulmonary disease; URTI, upper respiratory tract infection; LRTI, lower respiratory tract infection; ICU, intensive care unit; n.s., not significant; syst., systemic.

## Data Availability

Identified sequences were submitted to GenBank (accession no. OP927816 to OP928144).

## Supplementary Materials

The following supporting information can be downloaded at https://www.mdpi.com/article/10.3390/v15040877/s1; Table S1, Primers used for RSV sequencing; Table S2: Reagent composition for RSV-A sequencing (first PCR); Table S3: Reagent composition for RSV-B sequencing (first PCR); Table S4: Cycling conditions for RSV sequencing (first PCR); Table S5: Reagent composition for RSV-A sequencing (nested PCR); Table S6: Reagent composition for RSV-B sequencing (nested PCR); Table S7: Cycling conditions for RSV sequencing (nested PCR); Table S8: RSV-A reference sequences by Goya et al.; Table S9: RSV-A reference sequences by Muñoz-Escalante et al.; Table S10: RSV-B reference sequences by Goya et al.; Table S11: RSV-B reference sequences by Muñoz-Escalante et al.; Table S12: Co-infecting pathogens in the pediatric cohort; Table S13: Co-infecting pathogens in the pediatric cohort; Figure S1: Number of patients with laboratory-confirmed RSV infection; Figure S2: Molecular phylogenetic analysis of the RSV-A *G gene* using reference sequences proposed by Goya et al.; Figure S3: Molecular phylogenetic analysis of the RSV-A *G gene* using reference sequences proposed by Muñoz-Escalante et al.; Figure S4: Molecular phylogenetic analysis of the RSV-B *G gene* using reference sequences proposed by Goya et al.; Figure S5: Molecular phylogenetic analysis of the RSV-B *G gene* using reference sequences proposed by Muñoz-Escalante et al.
