# Supplementary material for: In-Depth Analysis of the Re-Emergence of Respiratory Syncytial Virus at a Tertiary Care Hospital in Germany in the Summer of 2021 after the Alleviation of Non-Pharmaceutical Interventions Due to the SARS-CoV-2 Pandemic
Source: Viruses. 2023 Mar 29;15(4):877. doi: 10.3390/v15040877 (PMC10144477; doi:10.3390/v15040877)
Supplement: Supplementary file 1 [file viruses-15-00877-s001.zip › viruses-2274213-supplementary.pdf]

In depth analysis of the re-emergence of respiratory syncytial virus in the summer of 2021 at a Tertiary Care Hospital in Germany after the alleviation of non-pharmaceutical interventions due to the SARS-CoV-2 pandemic

Mario Hönemann, Stephanie Thiem, Sandra Bergs, Tom Berthold, Christian Propach, Manuela Siekmeyer, Armin Frille, Tillmann Wallborn, Melanie Maier, and Corinna Pietsch

Supplementary Material

Table S1. Primers used for RSV sequencing.

Primer/Probe	Sequence (5´ to 3´)
RSV-A-F1	TCAAGCAAATTCTGGCCTTA
RSV-A-R1_mod#	CAACTGCAATTCTGTTACAGCA
RSV-A-F2	CCTTTGAGCTACCAAGAGCTC
RSV-A-R2	GAGTGTGACTGCAGCAAGGA
RSV-A-F3*	TGTCCGGAACTACATCACAATC
RSF-A-R3*	GCTGCATATGCTGCAGGG
RSV-A-Fseq*	CCCTGCAGCATATGCAGC
RSV-B-F1	ACAAGCAAATTTTGGCCCTA
RSV-B-R1	TAACTGTAATTCTGTTACTGCA
RSV-B-F2	CTCTTGAACAAGGACAGATGTATC
RSV-B-R2	CAATGCATTAATAGCAAGAG
RSV-B-F3*	CCAATCCACACAAACTCAGC
RSV-B-R3*	CACATATACTACAGGGAACAAAG
RSV-B-Fseq*	CTTTGTTCCCTGTAGTATATGTG

Primer concentration: 10 pmol/µl. # Modified from Ma et al. [1] * These primers were designed for the current study.

Table S2. Reagent composition for RSV-A sequencing (first PCR).

	Concentration	Volume [µl]	Final Concentration
**H_20**		20	
**5xBuffer**	5x (+12,5mM $MgCl_2$)	10	1x (+2,5mM $MgCl_2$)
**RSV-A-F1**	10 pmol/µl	3	30 pmol
**RSV-A-R1_mod**	10 pmol/µl	3	30 pmol
**dNTP's**	10 mM each	1	200 µM
**Enzyme-mix**		2	
**RNase Inhibitor**	40 Units/µl	1	40 Units
**RNA**		10	

Reagent concentrations and volumes used for RSV-A sequencing. The QIAGEN® OneStep RT-PCR Kit (Cat.No.210212) was used. Alternatively, in low viral load samples primers RSV-A-F1 & RSV-A-R3 and RSV-A-F3 & RSV-A-R1 were used to amplify smaller gene fragments.

Table S3. Reagent composition for RSV-B sequencing (first PCR).

	Concentration	Volume [µl]	Final Concentration
**H20**		20	
**5xBuffer**	5x (+12,5mM $MgCl_2$)	10	1x (+2,5mM $MgCl_2$)
**RSV-B-F1**	10 pmol/µl	3	30 pmol
**RSV-B-R1**	10 pmol/µl	3	30 pmol
**dNTP's**	10 mM each	1	200 µM
**Enzyme-Mix**		2	
**RNase Inhibitor**	40 Units/µl	1	40 Units
**RNA**		10	

Reagent concentrations and volumes used for RSV-B sequencing. The QIAGEN® OneStep RT-PCR Kit (Cat.No.210212) was used. Alternatively, in low viral load samples primers RSV-B-F1 & RSV-B-R3 and RSV-B-F3 & RSV-B-R1 were used to amplify smaller gene fragments.

Table S4. Cycling conditions for RSV-Sequencing (first PCR).

Reaction	Temperature	Time	Cycles
**Reverse transcription**	50°C	30 min	1
**Enzyme activation**	95°C	15 min	1
**Denaturation**	94°C	1 min	
**Annealing**	52°C	30 s	45
**Elongation**	72°C	1 min 30 s	
**Final elongation**	72°C	5 min	1
**Cooling**	4°C	forever	

The amplification was performed with a GeneTouch Thermal Cycler BTC33BAS (GeneTouch Corp., Taoyuan City, Taiwan).

Table S5. Reagent composition for RSV-A sequencing (nested PCR).

	Concentration	Volume [µl]	Final Concentration
**H_20**		33,1	
**10xBuffer,-$MgCl_2$**	10x	5	1x
**$MgCl_2$**	50 mM	1,5	1,5 MM
**RSV-A-F2**	10 pmol/µl	2	20 pmol
**RSV-A-R2**	10 pmol/µl	2	20 pmol
**dNTP's**	10 mM each	1	200 µM
**Platinum Taq**	5 Units/µl	0,4	2 Units
**First round PCR-product**		5	

Reagent concentrations and volumes used for RSV-A sequencing. The Invitrogen Platinum™ II Taq DNA Polymerase (Cat.No. 10966034) was used. Alternatively, in low viral load samples primers RSV-A-F2 & RSV-A-R3 and RSV-A-F3 & RSV-A-R2 were used to amplify smaller gene fragments.

Table S6. Reagent composition for RSV-B sequencing (nested PCR).

	Concentration	Volume [µl]	Final Concentration
**H_20**		33,1	
**10xBuffer,-$MgCl_2$**	10x	5	1x
**$MgCl_2$**	50 mM	1,5	1,5 MM
**RSV-B-F2**	10 pmol/µl	2	20 pmol
**RSV-B-R2**	10 pmol/µl	2	20 pmol
**dNTP's**	10 mM each	1	200 µM
**Platinum Taq**	5 Units/µl	0,4	2 Units
**First round PCR-product**		5	

Reagent concentrations and volumes used for RSV-A sequencing. The Invitrogen Platinum™ II Taq DNA Polymerase (Cat.No. 10966034) was used. Alternatively, in low viral load samples primers RSV- RSV-B-F2 & RSV-B-R3 and RSV-B-F3 & RSV-B-R2 were used to amplify smaller gene fragments.

Table S7. Cycling conditions for RSV-Sequencing (nested PCR).

Reaction	Temperature	Time	Cycles
**Enzyme activation**	95°C	1 min	1
**Denaturation**	94°C	30 s	45
**Annealing**	56°C	30 s	
**Elongation**	72°C	1 min 20 s	
**Final elongation**	72°C	5 min	1
**Cooling**	4°C	forever	

The amplification was performed on a GeneTouch Thermal Cycler BTC33BAS (GeneTouch Corp., Taoyuan City, Taiwan). The resulting PCR products were used for Sanger sequencing.

Table S8. RSV-A Reference Sequences Goya et al.

GA1	AY911262*	GA2.3.3	KJ627338	GA3.0.2	KU316104	GA2.3.5	KX765932
GA1	JX198138	GA2.3.3	KJ627349	GA3.0.2	KU316161	GA2.3.5	KX765941
GA1	KJ723474	GA2.3.3	KJ627370	GA3.0.2	KU316170	GA2.3.5	KX765954
GA1	KU316165	GA2.3.3	KJ939951	GA3.0.2	MG642048	GA2.3.5	KX765971
GA1	Z33427	GA2.3.3	KP317953	GA3.0.3a	AY910785	GA2.3.5	KX894807
GA2.1	KJ723483	GA2.3.3	KX655662	GA3.0.3a	JQ901455	GA2.3.5	KY654514
GA2.1	KP258723	GA2.3.3	KX655672	GA3.0.3a	JX069802	GA2.3.5	KY654518
GA2.1	KU316098	GA2.3.3	KX765958	GA3.0.3a	KF826847	GA2.3.5	KY883567
GA2.1	MG642070	GA2.3.3	KY654511	GA3.0.3a	KM360090	GA2.3.5	MG773271
GA2.2	JF920062	GA2.3.3	MF001041	GA3.0.3a	KY967364	GA2.3.6a	KT326808
GA2.2	JX069801	GA2.3.3	MF001047	GA3.0.3b	AY343561	GA2.3.6a	KT326810
GA2.2	KJ723492	GA2.3.3	MF001054	GA3.0.3b	AY910792	GA2.3.6a	KU350785
GA2.2	KP258700	GA2.3.4	JF920053	GA3.0.3b	JX513302	GA2.3.6a	KU350793
GA2.2	KP258743	GA2.3.4	KC731483	GA3.0.3b	JX513334	GA2.3.6a	KY634258
GA2.2	KU316092	GA2.3.4	KJ672455	GA3.0.4a	DQ985748	GA2.3.6b	KX453353
GA2.2	MG642030	GA2.3.4	KJ672482	GA3.0.4a	JX182792	GA2.3.6b	KX453385
GA2.3.0	JX069798	GA2.3.4	KP663728	GA3.0.4a	KF530260	GA2.3.6b	KX453442
GA2.3.0	KP119748	GA2.3.4	KU950667	GA3.0.4a	KF826854	GA2.3.6b	KX453518
GA2.3.0	KU316118	GA2.3.4	KX655658	GA3.0.4b	KF826826		
GA2.3.0	KU950573	GA2.3.4	KX765920	GA3.0.4b	KF826827		
GA2.3.0	MG642033	GA2.3.4	KY460517	GA3.0.4b	KF826850		
GA2.3.1	DQ985125	GA2.3.4	KY654508	GA3.0.4b	KF973333		
GA2.3.1	EU025215	GA2.3.4	MF001051	GA3.0.4b	KT765807		
GA2.3.1	JQ901452	GA2.3.4	MF001053	GA3.0.5b	JX645866		
GA2.3.1	JX015480	GA2.3.5	KF300977	GA3.0.5b	KC297367		
GA2.3.1	KJ627305	GA2	HQ731716	GA3.0.5b	KF826832		
GA2.3.1	MF496469	GA2	MG642063	GA3.0.5b	KX765933		
GA2.3.2a	AY343611	GA3.0.0	AF065406	GA3.0.5b	MF001038		
GA2.3.2a	KT765718	GA3.0.0	AF193308	GA2.3.5	JN257693		
GA2.3.2a	KT765726	GA3.0.0	AY343597	GA2.3.5	KJ672467		
GA2.3.2b	AY773299	GA3.0.0	DQ985132	GA2.3.5	KJ672470		
GA2.3.2b	JX015486	GA3.0.0	HQ731715	GA2.3.5	KT285064		
GA2.3.2b	KJ627284	GA3.0.0	KJ723486	GA2.3.5	KU950506		
GA2.3.2b	KJ627336	GA3.0.0	KP258709	GA2.3.5	KU950531		
GA2.3.2b	KU350846	GA3.0.0	KP258715	GA2.3.5	KU950540		
GA2.3.3	JX015482	GA3.0.0	KU316137	GA2.3.5	KU950550		
GA2.3.3	JX015491	GA3.0.0	KU316149	GA2.3.5	KU950556		
GA2.3.3	JX015497	GA3.0.0	MG642074	GA2.3.5	KU950560		
GA2.3.3	KF826838	GA3.0.1	KP258699	GA2.3.5	KU950596		
GA2.3.3	KF826855	GA3.0.1	KU316133	GA2.3.5	KU950650		
GA2.3.3	KJ627256	GA3.0.1	MG642031	GA2.3.5	KU950651		
GA2.3.3	KJ627294	GA3.0.2	KJ723465	GA2.3.5	KU950670		
GA2.3.3	KJ627320	GA3.0.2	KP258701	GA2.3.5	KU950692		
GA2.3.3	KJ627337	GA3.0.2	KP258726	GA2.3.5	KX765917		

Accession numbers of proposed genotypes [2]. *This sequence was used as RSV-A prototype strain sequence for the rooting of the phylogenetic trees throughout the manuscript.

Table S9. RSV-A Reference Sequences Muñoz-Escalante et al.

NA1	JX015495	GA3	Z33416
NA1	KC297260	GA3	Z33422
NA1	KC297277	GA3	Z33424
NA1	KC297292	GA3	Z33426
NA1	KF300972	GA5	AF065255
NA1	KP792358	GA5	AF193318
NA1	KP792359	GA5	AY114150
NA1	KF300973	GA5	AY114151
NA1	JN257694	GA5	AY343578
NA1	KP792361	GA5	AY343586
NA1	KP792362	GA5	HQ731701
NA1	KP792365	GA5	JX015487
NA1	KP792370	GA5	JX198135
NA1	KP792373	GA5	JX513382
NA1	KP792374	GA5	KJ627695
NA1	KP792375	GA5	KU316133
GA4	AF065254	GA5	Z33430
GA1	AF065257	GA5	Z33494
GA1	AF065407	GA6	DQ985131
GA1	AF233914	GA6	KP258723
GA1	JF920069	GA7	AF193320
GA1	KU316099	GA7	AF348804
GA1	KU316164	GA7	HQ731703
GA1	RSH1CE	GA7	X73351
GA1	RSHGLYG	GA7	Z33417
GA1	Z33427	GA7	Z33455
GA1	Z33431	SAA1	DQ985132
GA1	Z33432	SAA1	KP258696
GA2	AY114149	PRO	AY911262
GA2	HQ731710		
GA2	JQ901453		
GA3	AF065256		
GA3	AF193317		
GA3	AF193319		
GA3	AF233905		
GA3	AY343615		
GA3	AY343621		
GA3	DQ985122		
GA3	DQ985123		
GA3	KC297324		
GA3	KC297374		
GA3	KC297381		
GA3	MG642050		
GA3	Z33414		

Accession numbers of proposed genotypes [3]. Sequences encompassing only the 2nd hypervariable region of the G gene were used for the reconstruction of consensus sequences.

Table S10. RSV-B Reference Sequences Goya et al.

GB1	AY353550*	GB5.0.2	HQ731688	GB5.0.4b	KF826860
GB1	HQ731711	GB5.0.2	JX576742	GB5.0.4b	KJ627285
GB1	HQ731714	GB5.0.2	KC297492	GB5.0.4c	JX489429
GB1	JX198143	GB5.0.2	KF826843	GB5.0.4c	JX976333
GB1	KP258736	GB5.0.2	KF826845	GB5.0.4c	JX976378
GB1	M73545	GB5.0.2	KJ627302	GB5.0.4c	KC342326
GB2	AF013254	GB5.0.2	KJ939926	GB5.0.4c	KJ627262
GB2	HQ731708	GB5.0.2	KP862288	GB5.0.4c	KJ939928
GB2	HQ731718	GB5.0.2	KU950484	GB5.0.4c	KM402730
GB2	JX198165	GB5.0.2	KU950619	GB5.0.4c	KP317928
GB2	KP258712	GB5.0.2	KX655669	GB5.0.4c	KP862515
GB2	KU316127	GB5.0.2	KX765943	GB5.0.4c	KU950477
GB2	KU316173	GB5.0.2	KY249662	GB5.0.4c	KU950588
GB2	KU316175	GB5.0.3	JF704224	GB5.0.4c	KX655649
GB2	KU316181	GB5.0.3	JN032117	GB5.0.4c	KX655654
GB2	KU316182	GB5.0.3	JX576744	GB5.0.4c	KX765949
GB2	M73540	GB5.0.3	JX976394	GB5.0.4c	KY249658
GB2	MG642036	GB5.0.3	KC476971	GB5.0.4c	KY634397
GB2	MG642043	GB5.0.3	KF246627	GB5.0.4c	KY634410
GB3	AY333361	GB5.0.3	KF826839	GB5.0.4c	MF496628
GB3	JX198147	GB5.0.3	KM402680	GB5.0.4c	MG431251
GB3	JX198166	GB5.0.3	KM402697	GB5.0.5a	KX765906
GB3	M73543	GB5.0.3	KP862497	GB5.0.5a	KY249683
GB4	AF193331	GB5.0.3	KU950458	GB5.0.5a	KY684758
GB4	HQ731722	GB5.0.3	MG431252	GB5.0.5a	MG773268
GB4	JF704214	GB5.0.4a	KJ939929	GB5.0.5a	MG839547
GB4	JX198160	GB5.0.4a	KJ939932	GB5.0.5c	KX775755
GB4	MG642062	GB5.0.4a	KM402687	GB5.0.5c	KX775765
GB5.0.0	JX576760	GB5.0.4a	KU950467	GB5.0.5c	KX775767
GB5.0.0	KF826853	GB5.0.4a	KX655648	GB5.0.5c	KX775768
GB5.0.0	KP258713	GB5.0.4a	KX655653	GB6	JF704213
GB5.0.0	KP258724	GB5.0.4a	KX765912	GB6	KP862062
GB5.0.0	KP317923	GB5.0.4a	KX765957	GB6	KP862078
GB5.0.0	KU316134	GB5.0.4a	KX765962	GB6	KP862095
GB5.0.0	KU316179	GB5.0.4a	KY249657	GB6	MF185751
GB5.0.0	MF185754	GB5.0.4a	KY249670	GB7	KC297428
GB5.0.1	AY751111	GB5.0.4a	KY249677	GB7	KC297446
GB5.0.1	DQ227364	GB5.0.4a	KY883571	GB7	KC297462
GB5.0.1	JX576761	GB5.0.4b	JN032115	GB7	KC297466
GB5.0.1	JX576762	GB5.0.4b	JX576730	GB7	KF246637
GB5.0.1	KF826829	GB5.0.4b	JX576746	GB7	KT781406
GB5.0.1	KJ939919	GB5.0.4b	JX576751	GB7	MF496621
GB5.0.1	MF185752	GB5.0.4b	JX976389		
GB5.0.1	MF496543	GB5.0.4b	KF300963		

Accession numbers of proposed genotypes [2]. *This sequence was used as RSV-B prototype strain sequence for the rooting of the phylogenetic trees throughout the manuscript.

Table S11. RSV-B Reference Sequences Muñoz-Escalante et al.

BA	AB470481	BA	KF246585	BA	KT781399	GB2	DQ171865	GB3	DQ227407	PRO	RSHGLYGA
BA	AB470482	BA	KF246586	BA	KT781401	GB2	KP258720	GB3	DQ227408	SAB1	AF348825
BA	AB603467	BA	KF246607	BA	KU950587	GB2	KU316094	GB3	DQ270228	SAB1	AY524573
BA	AB603469	BA	KF246624	BA	KU950635	GB2	KU316156	GB3	DQ985154	SAB1	AY660682
BA	AB603470	BA	KF246629	BA	KU950682	GB3	AB175819	GB3	HM459858	SAB1	AY751272
BA	AB603476	BA	KF300952	BA	KX262619	GB3	AB175820	GB3	HM459860	SAB1	JF704213
BA	AB603477	BA	KF300953	BA	KX262621	GB3	AB175821	GB3	HM459861	SAB1	KP317939
BA	AY751087	BA	KF300954	BA	KX262625	GB3	AB603480	GB3	HM459863	SAB2	AF309676
BA	AY751093	BA	KF300955	BA	KX262638	GB3	AB603482	GB3	JX576761	SAB2	AF309678
BA	AY751273	BA	KF300957	BA	KX371866	GB3	AB603483	GB3	KP258702	SAB2	AF348821
BA	DQ227395	BA	KF300958	BA	KX371867	GB3	AB603484	GB3	KP258724	SAB2	AY327815
BA	DQ985136	BA	KF300959	BA	KX371868	GB3	AF233929	GB3	KP258742	SAB4	DQ270231
BA	DQ985143	BA	KF300960	BA	KX655648	GB3	AF233932	GB3	KP258745	SAB4	JN119976
BA	EU635867	BA	KF300970	BA	KX655669	GB3	AF233933	GB3	KU316100	SAB4	JN119979
BA	HM459864	BA	KF437511	BA	KX655681	GB3	AF348811	GB4	AF233924	SAB4	JN119987
BA	HM459865	BA	KF437512	BA	KX655686	GB3	AF348812	GB4	AF233928	SAB4	JN119989
BA	HM459866	BA	KF826822	BA	KX765959	GB3	AF348813	GB4	AF233931	SAB4	JN120007
BA	HM459867	BA	KJ690605	BA	KX775802	GB3	AF348817	GB4	AF348824	SAB4	KC297430
BA	HM459868	BA	KJ939929	BA	LC385001	GB3	AY333364	GB4	AY672691	SAB4	KC297478
BA	HM459870	BA	KM586843	BA	MF443156	GB3	AY488804	GB4	AY672698	URU2	AY333361
BA	HM459871	BA	KP336523	BA-C	KC297456	GB3	AY488805	GB4	AY751245	URU2	AY488803
BA	HM459872	BA	KP336524	BA-C	KC297486	GB3	AY751105	GB4	AY751246		
BA	HM459875	BA	KP336525	BA-CC	KU254638	GB3	AY751111	GB4	HQ731722		
BA	HM459878	BA	KP336526	BA-CC	KU254641	GB3	AY751116	GB4	JX198144		
BA	HM459880	BA	KP336527	BA-CC	KU254642	GB3	AY751117	GB4	KJ723484		
BA	HM459881	BA	KP336528	BA-CC	KU254643	GB3	AY751119	GB6	AY751237		
BA	HM459882	BA	KP336530	BA-CC	KX775822	GB3	AY751120	GB6	AY751239		
BA	HM459883	BA	KP336531	BA-CC	LC385004	GB3	AY751121	GB6	AY751241		
BA	HM459884	BA	KP336532	BA-CC	LC385008	GB3	AY751122	JAB1-NZB2	AB161386		
BA	HM459886	BA	KP336533	BA-CC	MF001058	GB3	AY751123	JAB1-NZB2	AB161387		
BA	HM459888	BA	KP336534	CB1-THB	KC297428	GB3	AY751150	JAB1-NZB2	AB161388		
BA	HM459890	BA	KP336535	CB1-THB	KC297471	GB3	AY751163	JAB1-NZB2	AB161389		
BA	HM459891	BA	KP336536	CB1-THB	KC342336	GB3	AY751174	JAB1-NZB2	AB161390		
BA	HQ731707	BA	KP336537	CB1-THB	KC342343	GB3	AY751228	JAB1-NZB2	AB161391		
BA	JX198152	BA	KP336538	GB1	AF013254	GB3	AY751230	JAB1-NZB2	AB161392		
BA	JX256976	BA	KP336540	GB1	AF065250	GB3	DQ171867	JAB1-NZB2	AB161395		
BA	JX256977	BA	KP336541	GB1	AY751256	GB3	DQ171878	JAB1-NZB2	AB161399		
BA	JX489454	BA	KP336542	GB1	KY674983	GB3	DQ227363	JAB1-NZB2	DQ171841		
BA	JX489456	BA	KP336543	GB1	RSHWV4843	GB3	DQ227364	JAB1-NZB2	DQ171842		
BA	JX645880	BA	KP336544	GB1	RSHWV10010	GB3	DQ227368	JAB1-NZB2	DQ171843		
BA	JX645887	BA	KP336545	GB1	RSHWV15291	GB3	DQ227370	JAB1-NZB2	DQ171844		
BA	JX645926	BA	KP862155	GB2	AF065251	GB3	DQ227373	JAB1-NZB2	DQ171845		
BA	JX908845	BA	KP862159	GB2	AY751250	GB3	DQ227374	JAB1-NZB2	DQ171846		
BA	KC297426	BA	KP862175	GB2	AY751280	GB3	DQ227375	JAB1-NZB2	DQ171847		
BA	KC297452	BA	KP862390	GB2	AY751281	GB3	DQ227377	JAB1-NZB2	HQ731709		
BA	KC297473	BA	KP862424	GB2	DQ171849	GB3	DQ227389	JAB1-NZB2	KJ723485		
BA	KC297476	BA	KR816639	GB2	DQ171858	GB3	DQ227393	JAB1-NZB2	KP258738		
BA	KC297484	BA	KR816642	GB2	DQ171862	GB3	DQ227396	JAB1-NZB2	KU316115		
BA	KC297492	BA	KR816654	GB2	DQ171863	GB3	DQ227397	JAB1-NZB2	KU316136		
BA	KC476943	BA	KT781395	GB2	DQ171864	GB3	DQ227403	PRO	JX198143		

Accession numbers of proposed genotypes [4]. Sequences encompassing only the 2nd hypervariable region of the G gene were used for the reconstruction of consensus sequences.

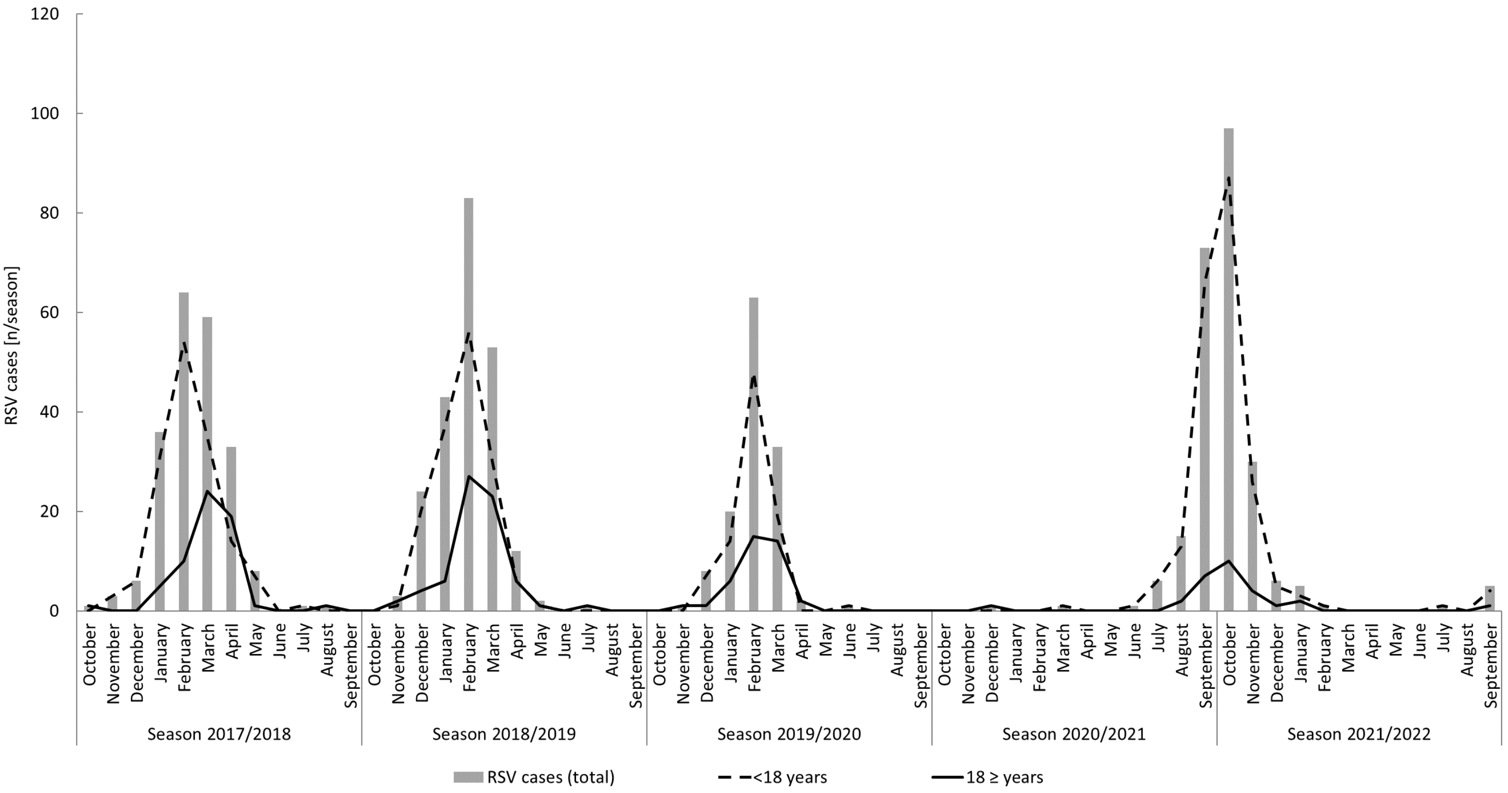

Supplementary Figure S1. Number of patients with laboratory-confirmed RSV infection. The dotted line represents the case numbers for patients under 18 years of age and the dashed line represents patients that were 18 years of age and older.

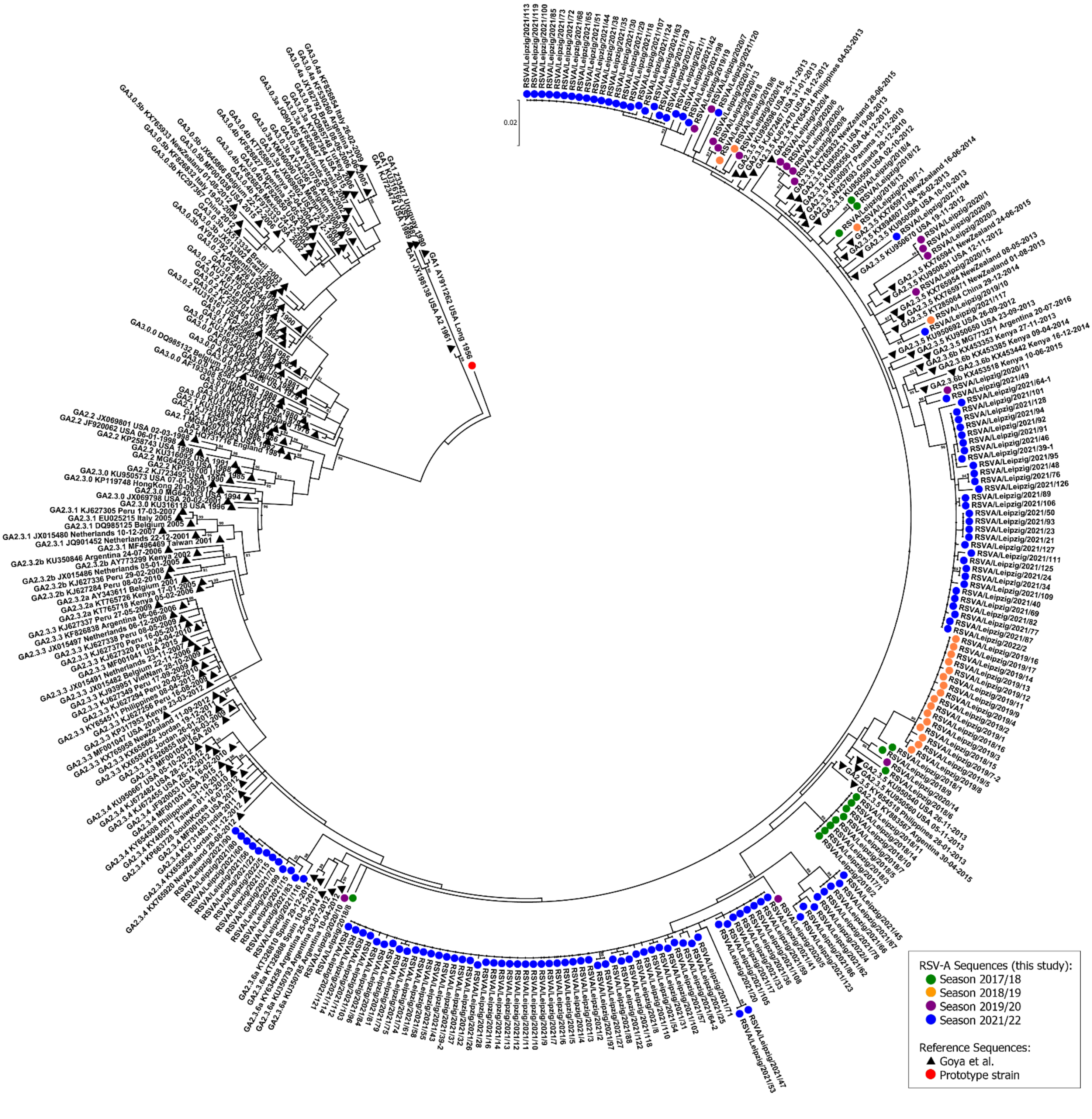

Supplementary Figure S2. Molecular Phylogenetic analysis of the RSV-A G gene using reference sequences proposed by Goya et al. [2]. The evolutionary history was inferred by using the Maximum Likelihood method based on the Tamura-Nei model [5]. The tree with the highest log likelihood (-8718.01) is shown. Initial tree(s) for the heuristic search were obtained automatically by applying Neighbor-Join and BioNJ algorithms to a matrix of pairwise distances estimated using the Maximum Composite Likelihood (MCL) approach, and then selecting the topology with superior log likelihood value. The tree is drawn to scale, with branch lengths measured in the number of substitutions per site. The analysis involved 335 nucleotide sequences. There were a total of 660 positions in the final dataset. Evolutionary analyses were conducted in MEGA7 [6]. Only nodes with a statistical support >80% are shown. The symbols indicate the sequence origin or the season of the indicated strain (dots: red, RSV-A reference strain; green: season 2017/2018 isolates; orange, season 2018/2019 isolates; purple, season 2019/2020 isolates; blue, season 2021/2022 isolates; black triangle: reference sequences).

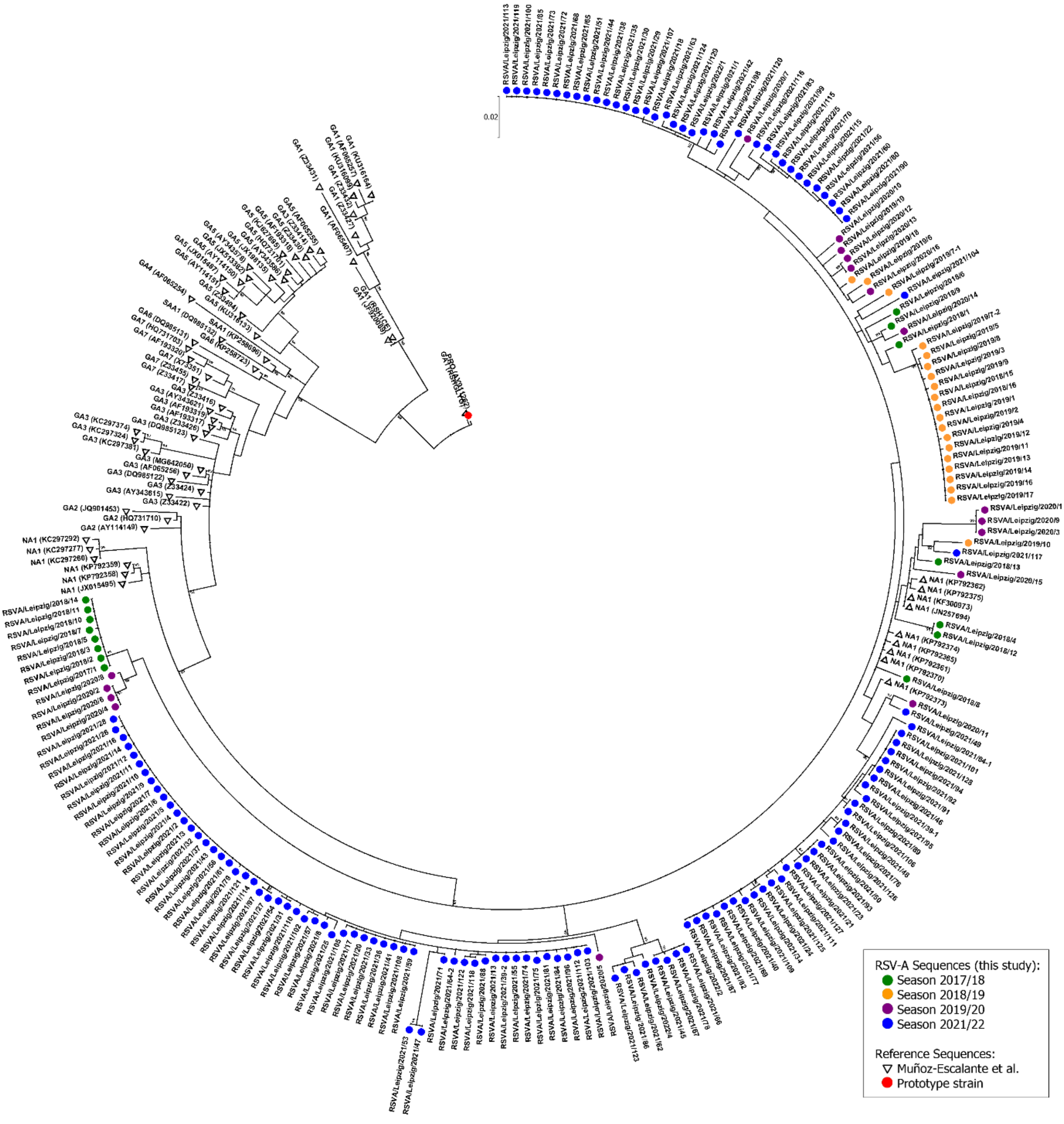

Supplementary Figure S3. Molecular Phylogenetic analysis of the RSV-A G gene using reference sequences proposed by Muñoz-Escalante et al. [3]. The evolutionary history was inferred by using the Maximum Likelihood method based on the Tamura-Nei model [5]. The tree with the highest log likelihood (-6868.83) is shown. Initial tree(s) for the heuristic search were obtained automatically by applying Neighbor-Join and BioNJ algorithms to a matrix of pairwise distances estimated using the Maximum Composite Likelihood (MCL) approach, and then selecting the topology with superior log likelihood value. The tree is drawn to scale, with branch lengths measured in the number of substitutions per site. The analysis involved 254 nucleotide sequences. There were a total of 698 positions in the final dataset. Evolutionary analyses were conducted in MEGA7 [6]. Only nodes with a statistical support >80% are shown. The symbols indicate the sequence origin or the season of the indicated strain (dots: red, RSV-A reference strain; green: season 2017/2018 isolates; orange, season 2018/2019 isolates; purple, season 2019/2020 isolates; blue, season 2021/2022 isolates; white triangle: reference sequences).

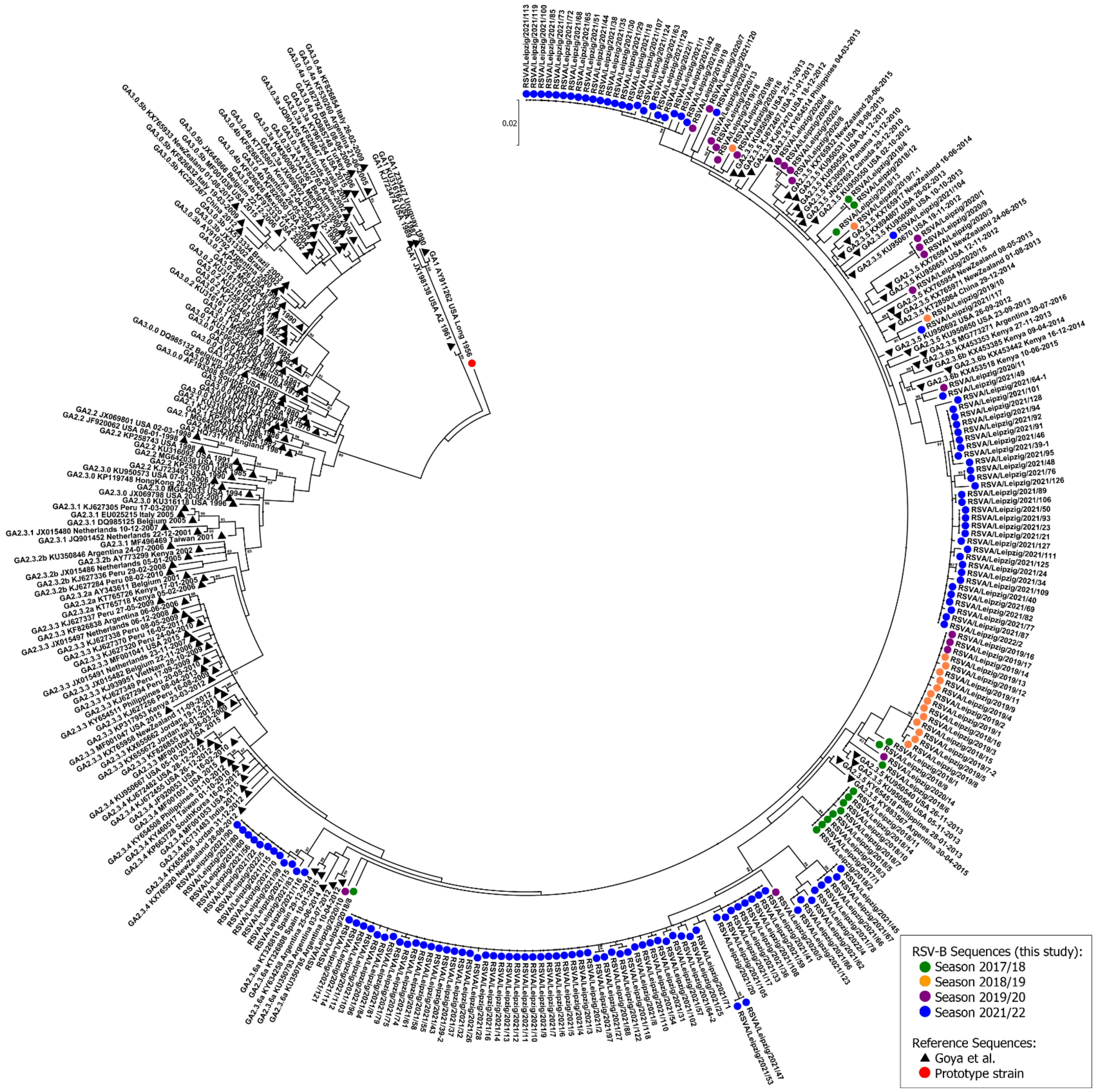

Supplementary Figure S4. Molecular Phylogenetic analysis of the RSV-B G gene using reference sequences proposed by Goya et al. [2]. The evolutionary history was inferred by using the Maximum Likelihood method based on the Tamura-Nei model [5]. The tree with the highest log likelihood (-7067.01) is shown. Initial tree(s) for the heuristic search were obtained automatically by applying Neighbor-Join and BioNJ algorithms to a matrix of pairwise distances estimated using the Maximum Composite Likelihood (MCL) approach, and then selecting the topology with superior log likelihood value. The tree is drawn to scale, with branch lengths measured in the number of substitutions per site. The analysis involved 274 nucleotide sequences. There were a total of 627 positions in the final dataset. Evolutionary analyses were conducted in MEGA7 [6]. Only nodes with a statistical support >80% are shown. The symbols indicate the sequence origin or the season of the indicated strain (dots: red, RSV-B reference strain; green: season 2017/2018 isolates; orange, season 2018/2019 isolates; purple, season 2019/2020 isolates; blue, season 2021/2022 isolates; black triangle: reference sequences).

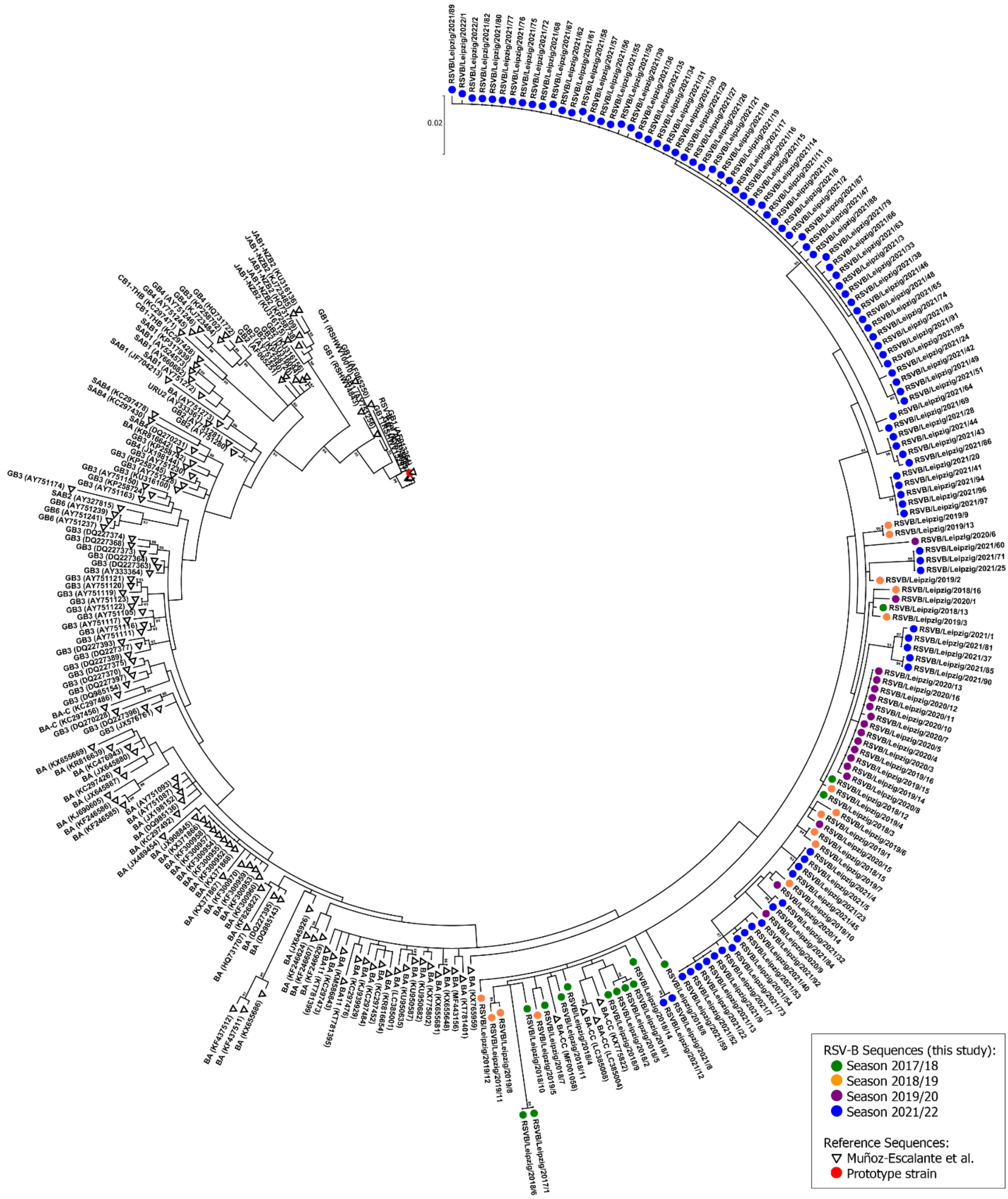

Supplementary Figure S5. Molecular Phylogenetic analysis of the RSV-B G gene using reference sequences proposed by Muñoz-Escalante et al. [4]. The evolutionary history was inferred by using the Maximum Likelihood method based on the Tamura-Nei model [5]. The tree with the highest log likelihood (-7688.68) is shown. Initial tree(s) for the heuristic search were obtained automatically by applying Neighbor-Join and BioNJ algorithms to a matrix of pairwise distances estimated using the Maximum Composite Likelihood (MCL) approach, and then selecting the topology with superior log likelihood value. The tree is drawn to scale, with branch lengths measured in the number of substitutions per site. The analysis involved 285 nucleotide sequences. There were a total of 745 positions in the final dataset. Evolutionary analyses were conducted in MEGA7 [6]. Only nodes with a statistical support >80% are shown. The symbols indicate the sequence origin or the season of the indicated strain (dots: red, RSV-B reference strain; green: season 2017/2018 isolates; orange, season 2018/2019 isolates; purple, season 2019/2020 isolates; blue, season 2021/2022 isolates; white triangle: reference sequences).

Table S12. Co-infecting pathogens in the pediatric cohort.

bacteria	*n*	viruses	*n*	fungi	*n*
Haemophilus influenzae	22	Bocavirus	46	*Candida albicans*	4
Moraxella catarrhalis	16	Rhinovirus	45	*Pneumocystis jirovecii*	1
Streptococcus pneumoniae	10	Adenovirus	23		
Staphylococcus aureus	7	Enterovirus	21		
Escherichia coli	6	Influenza A H1N1	8		
Klebsiella pneumoniae	5	Metapneumovirus	8		
Mycoplasma pneumoniae	5	Coronavirus NL63	6		
Pseudomonas aeruginosa	5	Coronavirus HKU1	5		
Enterobacter cloacae	4	Influenza B	5		
Enterobacter hormaechei	2	Coronavirus OC43	4		
Klebsiella oxytoca	2	Parainfluenzavirus Type 3	4		
Serratia marcescens	2	Coronavirus 229E	3		
Stapyhlococcus dysgalactiae	2	Influenza A H3N2	3		
Streptococcus pyogenes	2	EBV	2		
Bordetella parapertussis	1	Parainfluenzavirus Type 4	2		
Bordetella pertussis	1	CMV	1		
Chlamydophila pneumoniae	1	Parainfluenzavirus Type 1	1		
Haemophilus parahaemolyticus	1	SARS-CoV-2	1		
Leclercia adecarboxylata	1				
Proteus mirabilis	1				

Pathogens detected by type, with n showing their frequencies of detection. The co-infections included cases with detections of more than one pathogen.

Table S13. Co-infecting pathogens in the adult cohort.

**bacteria**	***n***	**viruses**	***n***	**fungi**	***n***
Pseudomonas aeruginosa	7	Influenza A H3N2	6	*Aspergillus fumigatus*	2
Klebsiella pneumoniae	6	Parainfluenzavirus Type 3	5	*Aspergillus niger*	2
Staphylococcus aureus	2	Metapneumovirus	3	*Pneumocystis jirovecii*	1
Escherichia coli	1	Rhinovirus	3		
Proteus mirabilis	1	Coronavirus OC43	2		
Raoultella ornithinolytica	1	Influenza B	2		
Serratia marcescens	1	SARS-CoV-2	2		
Streptococcus pneumoniae	1	Adenovirus	1		
		CMV	1		
		Dengue virus	1		
		Influenza A H1N1	1		
		Parainfluenzavirus Type 1	1		

Pathogens detected by type, with n showing their frequencies of detection. The co-infections included cases with detections of more than one pathogen.

References

1. Ma, Y.; Jiang, H.; Wan, Z.; Li, S.; Li, Y.; Wang, W.; Jin, X.; Li, Y.; Zhang, C. Evolutionary dynamics of group A and B respiratory syncytial virus in China, 2009-2018. *Arch. Virol.* **2021**, *166*, 2407–2418, doi:10.1007/s00705-021-05139-2.
2. Goya, S.; Galiano, M.; Nauwelaers, I.; Trento, A.; Openshaw, P.J.; Mistchenko, A.S.; Zambon, M.; Viegas, M. Toward unified molecular surveillance of RSV: A proposal for genotype definition. *Influenza Other Respir. Viruses* **2020**, *14*, 274–285, doi:10.1111/irv.12715.
3. Muñoz-Escalante, J.C.; Comas-García, A.; Bernal-Silva, S.; Robles-Espinoza, C.D.; Gómez-Leal, G.; Noyola, D.E. Respiratory syncytial virus A genotype classification based on systematic intergenotypic and intragenotypic sequence analysis. *Sci. Rep.* **2019**, *9*, 20097, doi:10.1038/s41598-019-56552-2.
4. Muñoz-Escalante, J.C.; Comas-García, A.; Bernal-Silva, S.; Noyola, D.E. Respiratory syncytial virus B sequence analysis reveals a novel early genotype. *Sci. Rep.* **2021**, *11*, 3452, doi:10.1038/s41598-021-83079-2.
5. Tamura, K.; Nei, M. Estimation of the number of nucleotide substitutions in the control region of mitochondrial DNA in humans and chimpanzees. *Mol. Biol. Evol.* **1993**, *10*, 512–526, doi:10.1093/oxfordjournals.molbev.a040023.
6. Kumar, S.; Stecher, G.; Tamura, K. MEGA7: Molecular Evolutionary Genetics Analysis Version 7.0 for Bigger Datasets. *Mol. Biol. Evol.* **2016**, *33*, 1870–1874, doi:10.1093/molbev/msw054.
